# Validation of Machine Learning-Based Individualized Treatment for Depressive Disorder Using Target Trial Emulation

**DOI:** 10.3390/jpm11121316

**Published:** 2021-12-07

**Authors:** Chi-Shin Wu, Albert C. Yang, Shu-Sen Chang, Chia-Ming Chang, Yi-Hung Liu, Shih-Cheng Liao, Hui-Ju Tsai

**Affiliations:** 1 National Centre for Geriatrics and Welfare Research, National Health Research Institutes, Zhunan 350, Taiwan; 2 Department of Psychiatry, Yunlin Branch, National Taiwan University Hospital, Yunlin 632, Taiwan; 3 Digital Medicine Center, Institute of Brain Science, National Yang-Ming Chiao-Tung University, Taipei 112, Taiwan; accyang@gmail.com; 4 Institute of Health Behaviours and Community Sciences, College of Public Health, National Taiwan University, Taipei 112, Taiwan; shusen.chang@gmail.com; 5 Department of Psychiatry, Chang Gung Memorial Hospital, Linkou and Chang Gung University, Taoyuan 333, Taiwan; cmchang58@yahoo.com.tw; 6 Department of Mechanical Engineering, National Taiwan University of Science and Technology, Taipei 106, Taiwan; lyh@mail.ntust.edu.tw; 7 Department of Psychiatry, College of Medicine, National Taiwan University Hospital, National Taiwan University, Taipei 100, Taiwan; scliao@ntu.edu.tw; 8 Institute of Population Health Sciences, National Health Research Institutes, Zhunan 350, Taiwan; tsaihj@nhri.edu.tw

**Keywords:** anti-depressive agents, machine learning, precision medicine

## Abstract

This study aims to develop and validate the use of machine learning-based prediction models to select individualized pharmacological treatment for patients with depressive disorder. This study used data from Taiwan’s National Health Insurance Research Database. Patients with incident depressive disorders were included in this study. The study outcome was treatment failure, which was defined as psychiatric hospitalization, self-harm hospitalization, emergency visits, or treatment change. Prediction models based on the Super Learner ensemble were trained separately for the initial and the next-step treatments if the previous treatments failed. An individualized treatment strategy was developed for selecting the drug with the lowest probability of treatment failure for each patient as the model-selected regimen. We emulated clinical trials to estimate the effectiveness of individualized treatments. The area under the curve of the prediction model using Super Learner was 0.627 and 0.751 for the initial treatment and the next-step treatment, respectively. Model-selected regimens were associated with reduced treatment failure rates, with a 0.84-fold (95% confidence interval (CI) 0.82–0.86) decrease for the initial treatment and a 0.82-fold (95% CI 0.80–0.83) decrease for the next-step. In emulation of clinical trials, the model-selected regimen was associated with a reduced treatment failure rate.

## 1. Introduction

Depressive disorder is a common psychiatric disorder that imposes a heavy social and economic burden [1,2]. Antidepressant therapy is the standard treatment for depressive disorders [3]. However, the effects of antidepressant treatment are limited, with a response rate of approximately 50% or less [4]. Approximately 29% to 46% of patients fail antidepressant treatment and develop treatment-resistant depression [5]. Various antidepressants have been approved for the treatment of depression. Meta-analyses have demonstrated varying therapeutic effects of different antidepressants [6]. However, this information was obtained based on collective data from clinical trials. Treatment responses and adverse effects may widely vary with patient’s characteristics, including age, sex, underlying conditions, and biological factors [7].

Developing individualized treatment can improve treatment effectiveness. Several studies have attempted to identify the predictors and moderators of pharmacological treatment outcomes of depressive disorders, such as pharmacogenetic [8,9] and clinical [7,10] factors. However, no individual predictor could be used to select individualized treatments in clinical practice [11]. Simultaneous consideration of numerous factors is a potential way to develop individualized treatments [12]. Machine learning has the advantage of being able to process high-dimensional information and complex interactions between variables. Machine learning has been used to predict the severity of depression [13]. In addition, individualized treatments based on machine-learning algorithms have been developed for the treatment of schizophrenia [14] and diabetes mellitus [15].

### Aims of the Study

This study aims to develop prediction models for individualized pharmacological treatment of depressive disorders, including the initial and next-step treatments, if previous treatments failed. In addition, a clinical trial was conducted to estimate the effectiveness of individualized pharmacological treatment using a population-based database in Taiwan.

## 2. Methods

### 2.1. Data Source

Taiwan’s National Health Insurance (NHI) program was launched in 1995. In 2009, 99.8% of the Taiwanese population was enrolled in the NHI program. The claims database derived from the NHI program, the National Health Insurance Research Database (NHIRD), includes information about the beneficiaries’ demographic characteristics, medical contacts, ICD-9-CM diagnoses, and prescription records. The accuracy of clinical diagnosis for major depressive disorders and other psychiatric illnesses has been validated [16]. This study was approved by the Research Ethics Committee of the National Taiwan University Hospital.

### 2.2. Study Sample

Initially, we identified 1,853,382 patients with diagnoses of depressive disorders (ICD-9-CM codes 296.2, 296.3, 300.4, and 311) from the NHIRD between 2001 and 2013. Individuals aged below 20 or above 75 years and those diagnosed with schizophrenia, bipolar disorder, or dementia were excluded. A total of 745,356 patients were included in our analysis.

The unit of analysis in this study was a treatment episode defined as the start of administration of the first antidepressant agent for incident patients, or a change in treatment regimen when previous treatment was ineffective or intolerable. The index date of a treatment episode was the date of starting or changing the treatment regimen. Treatment episodes were divided into two scenarios: the initial treatment episodes for the incident patients and the next-step treatment episodes if the previous treatments failed, including the second treatment and all subsequent treatments. There were 715,246 first treatment episodes and 739,980 next-step treatment episodes. A flowchart for selection of the treatment episodes is shown in the Supplementary Methods and Supplementary Figure S1.

### 2.3. The Development of the Prediction Model

#### 2.3.1. Data Training and Testing

We randomly divided the patients into two sets: 80% in training set and 20% in test set. Data management, the application of the machine learning algorithms of the prediction models, and statistical analyses were performed using SAS 9.4 software (SAS Institute, Inc., Cary, NC, USA) and R 3.5.3 software [17].

#### 2.3.2. Study Outcome

The treatment was considered a failure in the following four conditions: (1) psychiatric hospitalizations; (2) self-harm hospitalizations (defined using ICD-9-CM codes E950-E959); (3) emergency department visits due to psychiatric problems (defined using ICD-9-CM codes 290-319); or (4) treatment regimen changes including switching to or adding another antidepressant, or augmenting with second-generation antipsychotics or mood stabilizers, which indicated that the current treatment regimen resulted in either inadequate treatment responses or intolerable adverse reactions. Treatment discontinuation or dose titration was not considered as treatment failure because dose titration is necessary for some antidepressants, and one of the common reasons for discontinuation is symptom improvement [18,19]. Each treatment episode duration was a one-year observation period or until treatment failure occurred, whichever occurred first.

### 2.4. Predictors

Potential predictors of antidepressant treatment responses included the patients’ demographic variables, clinical characteristics of depression, and comorbid medical and psychiatric conditions. In Taiwan, patients can directly visit medical specialists without primary care physicians’ referrals. Thus, the number of outpatient visits to the specialists was used to identify each patient’s health-seeking behavior and underlying physical conditions. Patients’ various medication history was also extracted. Overall, there were 125 variables regarding patient characteristics for the initial treatment and 153 variables for the next-step treatment episodes. The additional 28 variables of next-step treatment included the characteristics of the previous regimen, the interval between the index and the last treatment episode, and the number of failed treatments. The definitions and distributions of these variables are presented in Supplementary Table S1.

### 2.5. Model Development

The treatment episodes in the training set were divided into several subsets based on the treatments. For example, patients treated with fluoxetine as the initial treatment were used to develop fluoxetine-initiating prediction models. There were 16 antidepressant-specific prediction models for the initial treatment and 41 models for the next-step treatment (16 strategies for antidepressant switching, 16 strategies for antidepressant combinations, and 9 strategies for augmentations). We did not include all regimens as a variable in a single model because the interaction variables between regimen and predictors are too numerous to develop a model efficiently. Therefore, we developed treatment-specific prediction models for each regimen. The contribution of each predictor varied across the different treatment-specific prediction models.

We conducted a filter-based feature selection (Pearson’s correlation coefficient p) to shorten training times and enhance generalization by reducing overfitting. We then used Super Learner to develop treatment-specific prediction models [20]. Super Learner is a supervised learning method that uses a stacking process to determine the optimal weighted combination of a collection of base machine learning algorithms [20]. Super Learner can include many diverse prediction models and perform equally to or better than the best-performing base algorithm. In this study, we used 19 base algorithms and combined them using the Lawson–Hanson algorithm to generate a Super Learner (Table 1). This model was analyzed using the SuperLearner package (version 2.0-26) in R [21]. In addition, we established different prediction models using logistic regression, random forest, and support vector machine to compare with the models using the Super Learner ensemble.

### 2.6. Statistical Analysis for Evaluating the Prediction Models

#### Prediction Performance

The outcomes predicted by combining all treatment-specific models were compared with the observed outcome in the test set. The performance of combining prediction models was evaluated using the area under the curve (AUC) of receiver operator characteristic analyses and the corresponding 95% confidence intervals (CIs) were estimated using the Delong method [22].

### 2.7. Evaluating the Effectiveness of the Machine-Selected Agents in the Test Set

The developed treatment-specific prediction models estimated the treatment failure probability across all potential antidepressant regimens for each treatment episode in the test dataset. The antidepressant with the lowest probability was defined as the model-selected agent. For the next-step treatment, there were three strategies: switching, combination, and augmentation. The three best regimens were identified, with each regimen having the lowest failure rate for each strategy.

**Figure 1:**
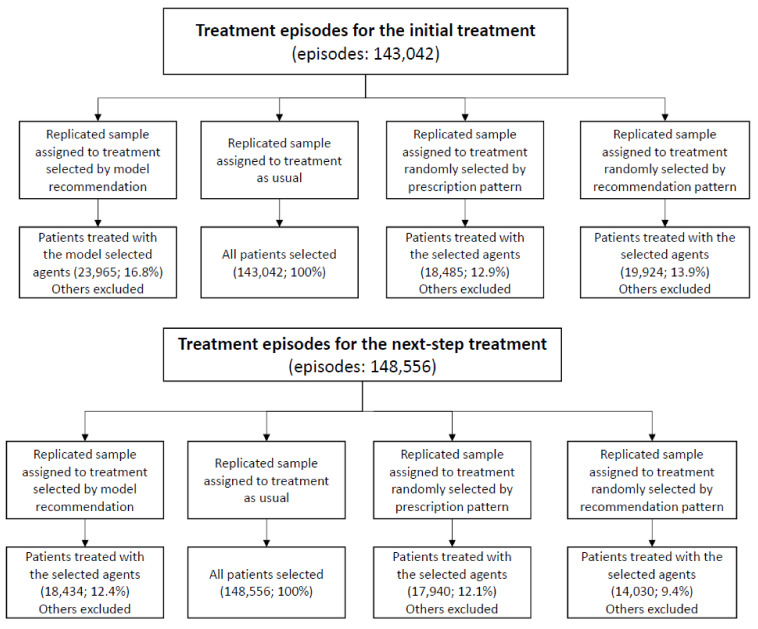
Study flowchart.

A randomized trial is the preferred design to evaluate the effect of the model-selected treatment. However, this is currently unavailable. Thus, the target trial was emulated [23,24] to estimate the effectiveness of the model-selected treatment. One target trial with four arms was considered, using two eligibility scenarios: (1) patients administered antidepressant treatment for depressive disorders for the first time or (2) patients just starting the next-step treatment if the previous ones failed. The four arms of trials for each scenario included the treatment group (treatment selected by model recommendation) and the three control groups (treatment as usual, treatment randomly selected based on the prescription proportions in the observed dataset, and treatment randomly selected based on the prescription proportion of the model recommendation). Four study samples were replicated to emulate the clinical trial. If the individual’s treatment was the same as the assigned treatment, the subject then would be enrolled into the arms (see Figure 1). All patients were followed up from their baseline visits and assessed every month until the study outcome occurred or the one-year follow-up period ended. A summary of the target trial protocol is listed in Supplementary Table S2.

We used intention-to-treat analysis and as-treated analysis to assess the effects of the model-selected regimen for the initial treatment and the next-step treatment. The details are described in the Supplementary Methods.

## 3. Results

A total of 715,246 patients with incident depressive disorders were included in the analysis. Among them, 283,927 patients received a next-step treatment, which led to 739,980 treatment episodes. The definition and distribution of baseline characteristics of the treatment episodes in the training and test datasets are shown in Supplementary Table S1.

The distribution and treatment failure rates of each prescribed antidepressant regimen in the training dataset are shown in Table 2. Overall, treatment failure rates varied markedly across antidepressant regimens. Regarding the initial antidepressant treatments, the overall treatment failure rate was 26.6%, ranging from 19.9% for imipramine to 30.9% for fluvoxamine. Regarding the next-step treatment episodes, the overall treatment failure rate was 54.3%, ranging from 48.5% for switching to escitalopram and 71.0% for switching to trazodone.

### Prediction Performance

The overall performance (quantified by AUC) of the combined initial treatment prediction model for treatment failure was 0.627 (95% CI: 0.623, 0.630), while the AUC of the overall next-step treatment prediction model was 0.751 (95% CI: 0.747, 0.756). The performance of the prediction models using the Super Learner ensemble was better than those using logistic regression, random forest, and support vector machine (Supplementary Table S3).

#### Evaluating the Effectiveness of the Machine-Selected Agents in the Test Set

Supplementary Table S4 shows the difference in the distributions of the observed and recommended treatments using the prediction model. The baseline characteristics of each treatment group for the initial treatment episode and the next-step treatment episodes in the emulating target trials are shown in Supplementary Tables S5 and S6, respectively.

Three pairwise comparisons of the treatment failure rates between the patients treated with the model-selected regimen and each of the three control groups are shown in Table 3. The model-selected regimens had the lowest failure rates across all treatment arms. In the ITT analyses of initial treatment episodes, the hazard ratios of the model-selected regimens showed a decrease in treatment failure by 0.84-fold (95% CI: 0.82, 0.86), 0.86-fold (95% CI: 0.82, 0.89), and 0.92-fold (95% CI: 0.89, 0.96) compared to treatment as usual, treatment randomly selected by the prescription proportion, and treatment randomly selected by the recommended proportion, respectively. Regarding the next-step treatment episodes, the hazard ratios of the model-selected treatment exhibited a decrease in treatment failure by 0.82-fold (95% CI: 0.80, 0.83), 0.85-fold (95% CI: 0.83, 0.88), and 0.87-fold (95% CI: 0.84, 0.90) compared with treatment as usual, treatment randomly selected by the prescription proportion, and treatment randomly selected by the recommended proportion, respectively. The results were similar to those of the as-treated analyses.

## 4. Discussion

### 4.1. Principal Results

This study used a machine learning approach to develop individualized pharmacological treatments for depression and estimated their effect by emulating clinical trials based on a large-scale database. The accuracy of the overall prediction models for treatment failure was 0.627 for the initial treatment episodes and 0.751 for the next-step treatment episodes. In addition, the model-selected regimens showed an 8–18% reduction in treatment failure rates compared with treatment as usual or when given randomly selected treatment in proportion to the actual or recommended treatments.

The model-selected treatment was associated with reduced treatment failure rates, with a moderate 8–18% decrease in treatment failure rates. However, individualized treatment strategies can generate large benefits across the population, as depression is a common mental illness. Furthermore, these prediction models were constructed based on the patients’ demographic variables, medical and psychiatric comorbid conditions, concomitant use of medications, and history of antidepressant treatment, which were obtained from the claims database. Thus, these prediction models can be easily implemented in Taiwan’s clinical electronic information system. Moreover, the prediction models can generate a list of antidepressant regimens according to their probabilities of treatment failure. Physicians then can prescribe agents with relatively low failure probabilities, if the first or previous agent was ineffective for the patient.

The distributions of the model-selected regimens were different from the observed prescription patterns. Although the model-selected treatment had a lower predicted treatment failure rate, this does not imply that the commonly recommended drugs are better than other drugs. The model recommendation was based on individual patients’ personal characteristics. Thus, the best treatment choice varies across individual patients. Furthermore, the distributions might differ when different patient populations are considered.

### 4.2. Limitations

The findings of this study should be interpreted with caution. First, common measures of treatment response in patients, such as symptom severity, functional level, or quality of life, were not directly assessed. Treatment changes, emergency visits, and hospitalizations were assumed either as poor treatment effectiveness or intolerance to adverse effects. However, these indicators may be insensitive and can only identify the worst conditions. Second, treatment episodes were used as the basic units of analysis. Therefore, poor responders with multiple treatment episodes were over-represented in the analysis for the next-step treatment. However, there is limited evidence for the best strategies for managing treatment-resistant depression. The prediction models of this study might provide a novel approach for improving treatment outcomes in this patient population. Finally, the claims data did not include several important variables, such as the reason for the treatment changes, socioeconomic factors, patient preferences, medication adherence, pharmacogenomics, or other biological markers. In addition, we used filter-based feature selection by Pearson’s correlation, which might be inadequate because some variables were not normally distributed. Therefore, unmeasured factors or inadequate variable selection can decrease the accuracy of prediction models. On the other hand, unmeasured factors would confound the estimation of the effects of the model-selected treatment. Our study only demonstrated associations, rather than causal effects. Thus, prospective clinical trials are warranted to validate the effectiveness of prediction models for individualized treatment.

### 4.3. Implications

The prediction models might be further optimized as clinical records accumulate and information on other relevant factors becomes available for model development. After a new antidepressant enters the market, data for its treatment responses could be rapidly collected in a population-based claims database, and its effectiveness can be assessed using the individualized treatment and prediction model strategy. Moreover, as large-scale genomic data and brain imaging information become more widely available, a machine-learning algorithm could integrate these data to improve the prediction accuracy of treatment response.

## 5. Conclusions

The prediction models presented in this study used a machine learning approach based on data of patient characteristics and history of antidepressant treatments. The overall accuracy of the prediction model was moderate. However, the model-selected treatments demonstrated reduced treatment failure rates compared to the usual treatments. Future prospective clinical trials investigating the effectiveness of individualized pharmacological treatment strategies are warranted to validate our findings.

## Figures and Tables

**Table 1:** Algorithms included in the Super Learner.

Algorithm Description	R Functions in SuperLearner
Bayesian GLM	SL.bayesglm
Generalized additive model	SL.gam
Generalized linear model	SL.glm
Ridge	SL.glmnet (alpha = 0)
Elastic net	SL.glmnet (alpha = 0.25)
	SL.glmnet (alpha = 0.5)
	SL.glmnet (alpha = 0.75)
LASSO	SL.glmnet (alpha = 1)
Support vector machine	SL.ksvm
k-nearest neighbors	SL.kernelKnn
Linear discriminant analysis	SL.lda
Neural network	SL.nnet
Polynomial spline regression	SL.polymars
Random forest	SL.ranger
Extreme gradient boosting	SL.xgboost (max_depth = 1, shrinkage = 0.01)
	SL.xgboost (max_depth = 1, shrinkage = 0.1)
	SL.xgboost (max_depth = 2, shrinkage = 0.01)
	SL.xgboost (max_depth = 2, shrinkage = 0.1)
	SL.xgboost (max_depth = 4, shrinkage = 0.01)
	SL.xgboost (max_depth = 4, shrinkage = 0.1)

Hyperparameters were specified if they did not use the default value.

**Table 2:** Distribution of treatment and failure rate among training data sets.

	Failure Rate, Overall (%)	Treatment Change (%)	Psychiatric Hospitalization (%)	Emergency Room Visits (%)	Self-Harm (%)
First treatment episodes (n; column %)					
Overall (572,204, 100%)	26.6	25.1	2.3	0.8	1.1
Amitriptyline (2131; 0.4%)	21.5	21.1	0.6	0.5	0.7
Bupropion (19,286; 3.4%)	27.7	26.6	2.0	0.6	0.8
Citalopram (50,724; 8.9%)	27.9	26.4	2.1	1.0	1.2
Doxepin (1727; 0.3%)	22.5	22.2	0.8	0.3	0.5
Duloxetine (15,671; 2.7%)	28.3	27.3	1.9	0.6	1.0
Escitalopram (56,338; 9.8%)	25.7	24.2	2.2	0.7	1.1
Fluoxetine (128,062; 22.4%)	22.8	21.2	2.2	0.9	1.1
Fluvoxamine (15,644; 2.7%)	30.9	29.5	2.3	1.0	1.2
Imipramine (4696; 0.8%)	19.9	19.4	1.5	0.7	0.5
Milnacipran (2938; 0.5%)	34.6	32.9	3.4	1.2	1.3
Mirtazapine (41,442; 7.2%)	29.8	27.9	3.0	0.9	1.4
Moclobemide (14,684; 2.6%)	25.1	24.2	1.3	0.6	0.7
Paroxetine (72,822; 12.7%)	29.0	27.5	2.5	0.9	1.1
Sertraline (102,517; 17.9%)	25.9	24.6	2.0	0.7	1.1
Trazodone (3510; 0.6%)	23.7	22.7	3.1	1.3	0.9
Venlafaxine (40,012; 7.0%)	30.7	29.1	2.9	0.8	1.3
Next-step treatment episodes (n, column %)					
Overall (591,424; 100%)	54.2	52.7	5.1	2.0	2.4
Switching to (538,050; 91.0%)	54.1	52.7	4.7	1.9	2.3
Amitriptyline (2926; 0.5%)	62.4	61.7	3.6	2.1	3.8
Bupropion (28,526; 4.8%)	58.5	57.4	4.4	1.3	1.8
Citalopram (44,037; 7.4%)	54.5	53.3	3.5	2.0	2.1
Doxepin (2692; 0.5%)	64.5	63.6	5.5	2.5	2.9
Duloxetine (24,485; 4.1%)	56.6	55.1	5.9	1.6	2.4
Escitalopram (60,965; 10.3%)	48.5	47.2	3.7	1.5	2.0
Fluoxetine (78,423; 13.3%)	49.6	48.0	4.5	2.1	2.4
Fluvoxamine (17,068; 2.9%)	59.3	57.9	5.5	2.1	2.5
Imipramine (4050; 0.7%)	63.1	62.0	5.0	2.1	2.1
Milnacipran (4963; 0.8%)	64.5	62.6	7.2	2.9	2.3
Mirtazapine (60,339; 10.2%)	60.5	58.7	6.3	2.2	3.0
Moclobemide (10,331; 1.7%)	52.0	51.1	3.7	1.4	1.5
Paroxetine (62,149; 10.5%)	54.0	52.6	4.6	1.9	2.2
Sertraline (77,362; 13.1%)	49.5	48.4	3.4	1.5	1.8
Trazodone (6030; 1.0%)	71.0	68.7	10.4	5.4	3.3
Venlafaxine (53,704; 9.1%)	56.8	55.1	5.7	1.9	2.6
Combinations with (n, column %) Overall (16,846; 2.8%)	55.6	53.3	8.7	2.5	3.2
Amitriptyline (478; 0.1%)	57.3	56.1	9.2	2.7	4.6
Bupropion (3475; 0.6%)	50.3	48.1	7.5	1.6	2.2
Citalopram (440; 0.1%)	58.0	56.1	7.3	4.1	5.5
Doxepin (421; 0.1%)	68.9	65.8	12.8	5.0	3.8
Duloxetine (873; 0.1%)	54.2	51.1	9.0	1.9	3.4
Escitalopram (1117; 0.2%)	54.1	51.9	8.6	1.7	3.2
Fluoxetine (1552; 0.3%)	54.1	52.1	7.8	2.8	3.5
Fluvoxamine (255; 0.0%)	65.1	61.6	8.6	3.9	5.1
Imipramine (599; 0.1%)	62.1	59.9	6.5	1.3	2.8
Milnacipran (174; 0.0%)	65.5	64.4	8.6	3.4	3.4
Mirtazapine (2577; 0.4%)	55.6	53.3	9.0	2.4	2.8
Moclobemide (168; 0.0%)	60.1	58.9	7.1	2.4	3.0
Paroxetine (964; 0.2%)	53.2	51.0	9.4	2.5	2.9
Sertraline (1037; 0.2%)	51.0	49.3	6.2	2.2	2.8
Trazodone (1148; 0.2%)	62.7	59.5	13.5	5.3	4.0
Venlafaxine (1568; 0.3%)	59.8	56.8	9.8	2.3	4.3
Augmentations (36,528; 6.2%)	55.7	52.4	10.2	2.8	3.6
Amisulpride (1644; 0.3%)	59.2	56.8	10.5	1.8	2.4
Aripiprazole (3499; 0.6%)	55.4	52.7	9.4	2.0	2.7
Olanzapine (2215; 0.4%)	64.0	61.0	13.1	2.6	3.7
Quetiapine (15,119; 2.6%)	54.3	50.8	9.8	2.9	3.9
Risperidone (2894; 0.5%)	55.6	52.3	11.0	3.1	2.4
Zotepine (1274; 0.2%)	67.1	62.9	14.4	3.3	5.8
Lamotrigine (1442; 0.2%)	57.4	54.4	9.8	2.1	3.5
Lithium (1839; 0.3%)	58.9	56.6	8.9	2.0	3.4
Valproic acid (6602; 1.1%)	52.1	48.2	9.7	3.5	4.1

**Table 3:** Treatment failure rates of the groups with treatment selected using model recommendation compared with the control groups.

	Intention-to-Treat Analysis		As-Treated Analysis	
	Incidence of treatment failure (no./person-year)	Hazard ratio (95% Confidence intervals) Treatment vs. controls	Incidence of treatment failure (no./person-year)	Hazard ratio (95% Confidence intervals) Treatment vs. controls
Initial treatment episodes				
Treatment selected by model recommendation	0.27 (5314/20,032)		0.39 (3811/9784)	
Treatment as usual	0.34 (38,289/114,250)	0.84 (0.82, 0.86)	0.47 (28,102/60,161)	0.88 (0.85, 0.91)
Treatment selected randomly by prescription proportion	0.32 (4812/14,879)	0.86 (0.82, 0.89)	0.45 (3470/7664)	0.87 (0.83, 0.91)
Treatment selected randomly by recommendation proportion	0.29 (4757/16,363)	0.92 (0.89, 0.96)	0.43 (3455/8098)	0.87 (0.83, 0.91)
Next-step treatment episodes				
Treatment selected by model recommendation	0.70 (8460/12,110)		0.89 (6389/7177)	
Treatment as usual	0.94 (80,478/85,742)	0.82 (0.80, 0.83)	1.26 (63,195/50,335)	0.82 (0.80, 0.85)
Treatment selected randomly by prescription proportion	0.91 (9528/10,519)	0.85 (0.83, 0.88)	1.22 (7482/6154)	0.85 (0.82, 0.88)
Treatment selected randomly by recommendation proportion	0.85 (7199/8472)	0.87 (0.84, 0.90)	1.12 (5560/4975)	0.82 (0.80, 0.84)

## Data Availability

Restrictions apply to the availability of some or all data generated or analyzed during this study to preserve patient confidentiality or because they were used under license. The corresponding author will request details of the restrictions and any conditions under which access to some data may be provided.

## Supplementary Materials

The following are available online at https://www.mdpi.com/article/10.3390/jpm11121316/s1, Figure S1: Flowchart of study cohort formation, Table S1: Baseline characteristics for the initial and next-step treatment episodes, Table S2: Study design for target trials, Table S3: Comparisons of the performance of the prediction models using Super Learner ensemble, logistic regression, random forest, and support vector machine, Table S4: Distribution of observed and recommended treatment among test datasets, Table S5: The baseline characteristics of these four groups for the initial treatment, Table S6: The baseline characteristics of these four groups for the next-step treatment.
