# Supplementary material for: Validation of Machine Learning-Based Individualized Treatment for Depressive Disorder Using Target Trial Emulation
Source: J Pers Med. 2021 Dec 7;11(12):1316. doi: 10.3390/jpm11121316 (PMC8706481; doi:10.3390/jpm11121316)
Supplement: Supplementary file 1 [file jpm-11-01316-s001.zip › Supplementary tables_1.pdf]

Supplementary Method

Study sample

The data were only extracted between 2001 and 2013 when the International Classification of Disease, Ninth Revision, Clinical Modification (ICD-9-CM) codes were fully applied. We identified 1,853,382 patients with diagnoses of depressive disorders (ICD-9-CM code: 296·2, 296·3, 300·4, and 311). The information for initial treatment was not available for prevalent patients. Therefore, only patients with newly diagnosed depressive disorders, namely incident patients, were included. A two-year washout period (year 2001 and 2002) was used to exclude prevalent patients (n=457,306). In order to have at least one-year follow-up period, we excluded patients with a first diagnosis of depressive disorder in 2013 (n=6,083). We further excluded patients whose age was below 20 or above 75 years (n=183,603), had missing sex information (n=2,151), or had a diagnosis of schizophrenia, bipolar disorder, or dementia prior to the first depression diagnosis (n=122,178). Patients not receiving antidepressant treatments (n=171,812) or being treated with inadequate doses (<0·5 defined daily dose [DDD][1]; n=164,893) were also excluded. Finally, there were 745,356 patients included in our analysis. These patients randomly divided into training (n=596,285) and test set (n=149,071).

The unit of analysis in this study was a treatment episode, which was defined as the start of administration of the first antidepressant agent for incident patients, or a change in treatment regimen. The index date of a treatment episode is the date of starting or changing of the treatment regimen. The treatment regimen could be monotherapy (antidepressant only) or polypharmacy (combination with another antidepressant or augmentation with a second-generation antipsychotic drug or a

mood stabiliser). If the change in regimen was a step-down strategy from polypharmacy to monotherapy without initiating a new antidepressant, this condition was not classified as a new treatment episode.

A total of 1,549,903 treatment episodes were identified. Treatment episodes were divided into two scenarios: the initial treatment episodes for the incident patients, and the next-step treatment episodes if the previous treatments failed, including the second treatment and all treatment after the second one. Common strategies for the next-step treatments included switching to another antidepressant if the previous regimen was ineffective or intolerant, combining the current medication with another antidepressant, or augmentation with second-generation antipsychotics or mood stabilisers if the previous antidepressant's effect was suboptimal and there were no severe adverse reactions [2,3]. Episodes including rarely used agents or initiating or changing two or more agents simultaneously were excluded. Those with combinations of three or more agents were also excluded. Finally, there were 715,246 first treatment episodes, including 572,204 episodes in training set ad 143,042 episodes; and 739,980 next-step treatment episodes, including 591,424 episodes in training set, and 148,556 in test set. A flowchart of the selection of the treatment episodes is shown in online Supplementary Fig. S1.

Statistical analysis in the emulating target trial

Intention-to-treat analysis

In order to emulate clinical trial analysis, the intention-to-treat effect of the model-selected regimen on treatment failure was estimated by pair-wise comparing with

the control groups. If the patients discontinued the treatment, they were still followed up until the end of the one-year observational period. The distribution of the baseline variables for these four arms are shown in supplementary Table S4. The effects of the model-selected regimen for the initial treatment and the next-step treatment were estimated separately.

In order to mimic the random assignment of strategies at baseline, the baseline variables were used to calculate each patient's propensity score of receiving each treatment.[4] Inverse probability weighting (IPW), the inverse of the probability of treatment (propensity score), was used to remove confounding by creating a 'pseudo-population' in which the treatment is independent of the measured confounders. The hazard ratio of the treated versus the control groups were estimated using Cox proportional hazards models with IPW method.[4]

As-treated analysis

Under actual circumstances, the antidepressant treatment might discontinue over the follow-up period. Therefore, the as-treated analyses were conducted to classify patients according to the treatment that they actually received during the follow-up period rather than the treatment they were assigned at baseline. In addition, the patients' covariate statuses might change over time and they might also drop out over the study period. Thus, marginal structured models were used to adjust time-varying covariates and estimate the function of censors [5]. In the marginal structured models, a time-variant IPW method was used to adjust for potential time-varying selection biases due to artificial censoring, as well as time-varying confounding. The time-variant IPW for each patient was calculated at each visit from

Month 0 (baseline) to Month 11. Based on the time-varying IPW, we generated a pseudo-population in which there was no association between the measured confounders and treatment at any time during the follow-up period. A censoring weighting was also performed to control for artificial censoring. Thus, the marginal structured modelling approach was able to eliminate both measured confounding and selection biases. All the covariates listed in supplementary Table S1 were assessed at each visit.

Supplementary Table S1. Baseline characteristics for the initial and next-step treatment episodes

	The initial treatment episodes			The next-step treatment episodes		
	Total (715,246)	Training (572,204)	Test set (143,042)	Total (739,980)	Training (591,424)	Test set (148,556)
Age group						
18-29	193,468 (27.0)	154,702 (27.0)	38,766 (27.1)	166,428 (22.5)	133,568 (22.6)	32,860 (22.1)
30-39	144,768 (20.2)	115,972 (20.3)	28,796 (20.1)	163,997 (22.2)	131,041 (22.2)	32,956 (22.2)
40-49	143,196 (20.0)	114,618 (20.0)	28,578 (20.0)	171,185 (23.1)	137,238 (23.2)	33,947 (22.9)
50-59	122,523 (17.1)	97,875 (17.1)	24,648 (17.2)	134,873 (18.2)	107,437 (18.2)	27,436 (18.5)
≥60	111,291 (15.6)	89,037 (15.6)	22,254 (15.6)	103,497 (14.0)	82,140 (13.9)	21,357 (14.4)
Sex, female	462,111 (64.6)	369,643 (64.6)	521,630 (64.6)	338,291 (68.4)	385,207 (68.4)	525,288 (68.4)
**Type of depression**						
Major depressive disorder	204,215 (28.6)	163,603 (28.6)	40,612 (28.4)	311,884 (42.1)	249,322 (42.2)	62,562 (42.1)
Dysthymic disorder	392,550 (54.9)	313,971 (54.9)	78,579 (54.9)	328,040 (44.3)	262,318 (44.4)	65,722 (44.2)
Depressive disorder, NOS	118,481 (16.6)	94,630 (16.5)	23,851 (16.7)	100,056 (13.5)	79,784 (13.5)	20,272 (13.6)
**Duration of illness**	0.2 ±0.9	0.2 ±0.9	0.2 ±1.0	2.5 ±2.3	2.5 ±2.3	2.5 ±2.3
**Comorbidity**						
Diabetes mellitus (ICD-9-CM: 250)	64,117 (9.0)	51,228 (9.0)	12,889 (9.0)	68,763 (9.3)	54,859 (9.3)	13,904 (9.4)
Dyslipidemia (ICD-9-CM: 272)	92,462 (12.9)	73,933 (12.9)	18,529 (13.0)	107,669 (14.6)	85,689 (14.5)	21,980 (14.8)
Asthma (ICD-9-CM: 493)	32,539 (4.5)	26,057 (4.6)	6,482 (4.5)	39,697 (5.4)	31,578 (5.3)	8,119 (5.5)
Cancer (ICD-9-CM: 140-208, 273.0, 273.3, and V10)	26,623 (3.7)	21,234 (3.7)	5,389 (3.8)	28,030 (3.8)	22,400 (3.8)	5,630 (3.8)
Headache (ICD-9-CM: 307.81, 346, 784.0)	216,029 (30.2)	172,475 (30.1)	43,554 (30.4)	282,145 (38.1)	225,878 (38.2)	56,267 (37.9)
Hemiplegia (ICD-9-CM: 342, 344)	7,955 (1.1)	6,349 (1.1)	1,606 (1.1)	6,854 (0.9)	5,588 (0.9)	1,266 (0.9)
Peripheral neurological disorder (ICD-9-CM: 351-357)	50,410 (7.0)	40,270 (7.0)	10,140 (7.1)	60,209 (8.1)	48,349 (8.2)	11,860 (8.0)
Cerebrovascular disease (ICD-9-CM: 362.34, 430-438, 781.4, 784.3, 997.0)	36,265 (5.1)	28,888 (5.0)	7,377 (5.2)	35,114 (4.7)	28,052 (4.7)	7,062 (4.8)
Hypertension (ICD-9-CM: 401-405)	142,053 (19.9)	113,415 (19.8)	28,638 (20.0)	150,703 (20.4)	119,760 (20.2)	30,943 (20.8)
Congestive heart failure (ICD-9-CM: 402.01, 402.11, 302.91, 425, 428, 429.3)	15,981 (2.2)	12,750 (2.2)	3,231 (2.3)	15,034 (2.0)	12,010 (2.0)	3,024 (2.0)
Chronic kidney disease (ICD-9-CM: 403.x1, 404.x2, 585, 586, V42.0, V45.1, V56.0, V56.8)	9,604 (1.3)	7,732 (1.4)	1,872 (1.3)	9,308 (1.3)	7,399 (1.3)	1,909 (1.3)
Coronary heart disease (ICD-9-CM: 410-414)	61,280 (8.6)	48,935 (8.6)	12,345 (8.6)	63,555 (8.6)	51,026 (8.6)	12,529 (8.4)

Peripheral vascular disease (ICD-9-CM: 440, 441.2, 441.4, 441.7, 441.9, 443.1- 443.9, 447.1, 557, 785.4)	10,152 (1.4)	8,088 (1.4)	2,064 (1.4)	11,272 (1.5)	8,938 (1.5)	2,334 (1.6)
Chronic pulmonary disease (ICD-9-CM: 491, 492, 494, 495, 496)	34,392 (4.8)	27,443 (4.8)	6,949 (4.9)	36,305 (4.9)	29,066 (4.9)	7,239 (4.9)
Peptic ulcer disease (ICD-9-CM: 531 – 534)	126,680 (17.7)	101,346 (17.7)	25,334 (17.7)	157,498 (21.3)	126,222 (21.3)	31,276 (21.1)
Functional gastrointestinal disorders (ICD-9-CM: 536, 564, 306.4)	242,680 (33.9)	194,300 (34.0)	48,380 (33.8)	320,616 (43.3)	257,007 (43.5)	63,609 (42.8)
Chronic liver disease (ICD-9-CM: 571.2-571.9; 456.0-456.2)	18,222 (2.5)	14,595 (2.6)	3,627 (2.5)	21,957 (3.0)	17,596 (3.0)	4,361 (2.9)
Rheumatological disease (ICD-9-CM: 710.0, 710.1, 710.4, 714.0, 714.1, 714.2, 714.81, 725)	9,873 (1.4)	7,906 (1.4)	1,967 (1.4)	11,893 (1.6)	9,500 (1.6)	2,393 (1.6)
Fibromyalgia and osteoarthritis (ICD-9-CM: 715, 729.1)	215,980 (30.2)	172,710 (30.2)	43,270 (30.2)	259,113 (35.0)	206,669 (34.9)	52,444 (35.3)
Back pain (ICD-9-CM: 721, 722, 723, 724, 739.3,739.4, 846, 847.2)	220,414 (30.8)	176,134 (30.8)	44,280 (31.0)	261,146 (35.3)	208,783 (35.3)	52,363 (35.2)
Alcohol use disorder (ICD-9-CM: 291, 303.9, 305.0, 357.5, 425.5, 535.3, 571.0-571.3, V11.3)	20,886 (2.9)	16,819 (2.9)	4,067 (2.8)	34,364 (4.6)	27,725 (4.7)	6,639 (4.5)
Substance use disorder (ICD-9-CM: 292, 304, 305.2-305.9)	6,952 (1.0)	5,572 (1.0)	1,380 (1.0)	22,787 (3.1)	18,249 (3.1)	4,538 (3.1)
Anxiety state, unspecified (ICD-9-CM: 300 without 4th or 5th digit)	8,293 (1.2)	6,624 (1.2)	1,669 (1.2)	8,295 (1.1)	6,724 (1.1)	1,571 (1.1)
Panic disorder (ICD-9-CM: 300.01)	31,710 (4.4)	25,347 (4.4)	6,363 (4.4)	69,721 (9.4)	55,446 (9.4)	14,275 (9.6)
Generalized anxiety disorder (ICD-9-CM: 300.02)	59,363 (8.3)	47,379 (8.3)	11,984 (8.4)	97,860 (13.2)	78,322 (13.2)	19,538 (13.2)
Phobic disorders (ICD-9-CM: 300.2)	5,830 (0.8)	4,720 (0.8)	1,110 (0.8)	11,856 (1.6)	9,513 (1.6)	2,343 (1.6)
Neurasthenia (ICD-9-CM: 300.5)	5,345 (0.7)	4,212 (0.7)	1,133 (0.8)	10,348 (1.4)	8,223 (1.4)	2,125 (1.4)
Personality disorders (ICD-9-CM: 301)	5,427 (0.8)	4,352 (0.8)	1,075 (0.8)	19,730 (2.7)	15,780 (2.7)	3,950 (2.7)
Adjustment disorders (ICD-9-CM: 309)	50,781 (7.1)	40,727 (7.1)	10,054 (7.0)	84,060 (11.4)	67,461 (11.4)	16,599 (11.2)
Obsessive-compulsive disorders (ICD-9-CM:300.3)	9,493 (1.3)	7,643 (1.3)	1,850 (1.3)	20,632 (2.8)	16,532 (2.8)	4,100 (2.8)
Somatoform disorders (ICD-9-CM:300.8)	9,557 (1.3)	7,602 (1.3)	1,955 (1.4)	17,414 (2.4)	13,995 (2.4)	3,419 (2.3)
Other anxiety disorders (ICD-9-CM:300.1, 300.6, 300.7, 300.9)	51,187 (7.2)	40,912 (7.1)	10,275 (7.2)	78,761 (10.6)	63,182 (10.7)	15,579 (10.5)
Physiological malfunction arising from mental factors (ICD-9-CM:306)	29,709 (4.2)	23,624 (4.1)	6,085 (4.3)	44,592 (6.0)	35,641 (6.0)	8,951 (6.0)
Sleep disorders (ICD-9-CM:307.4, 780.5)	333,418 (46.6)	266,629 (46.6)	66,789 (46.7)	459,982 (62.2)	367,308 (62.1)	92,674 (62.4)
Eating disorders (ICD-9-CM: 307.1, 307.5)	4,011 (0.6)	3,218 (0.6)	793 (0.6)	10,176 (1.4)	7,976 (1.3)	2,200 (1.5)
**Number of outpatient visits**						

Psychiatry	0.8 ±2.1	0.8 ±2.1	0.8 ±2.1	5.2 ±4.0	5.2 ±4.0	5.2 ±4.0
Family medicine	3.2 ±3.6	3.2 ±3.6	3.2 ±3.6	3.6 ±3.8	3.6 ±3.8	3.6 ±3.8
Internal medicine	2.8 ±3.5	2.8 ±3.5	2.8 ±3.5	3.5 ±3.8	3.5 ±3.8	3.4 ±3.8
Dermatology	0.8 ±1.8	0.8 ±1.8	0.8 ±1.8	0.9 ±1.9	0.9 ±1.9	0.9 ±2.0
Neurology	0.6 ±1.7	0.6 ±1.7	0.6 ±1.7	0.8 ±2.1	0.8 ±2.1	0.8 ±2.1
Obstetrics and gynecology	1.3 ±2.6	1.3 ±2.6	1.3 ±2.6	1.6 ±2.7	1.6 ±2.7	1.6 ±2.7
Ophthalmology	0.9 ±2.0	0.9 ±2.0	0.9 ±2.0	1.0 ±2.0	1.0 ±2.0	1.0 ±2.0
Orthopedics	0.7 ±1.8	0.7 ±1.8	0.7 ±1.8	0.9 ±2.1	0.9 ±2.1	0.9 ±2.1
Otolaryngology	1.6 ±2.6	1.6 ±2.6	1.6 ±2.6	1.8 ±2.8	1.8 ±2.8	1.8 ±2.8
Rehabilitation	0.4 ±1.5	0.4 ±1.5	0.4 ±1.5	0.5 ±1.7	0.5 ±1.7	0.5 ±1.6
Surgery	0.6 ±1.6	0.6 ±1.6	0.6 ±1.6	0.8 ±1.8	0.8 ±1.8	0.7 ±1.8
Other specialties	1.6 ±2.7	1.6 ±2.7	1.6 ±2.7	1.9 ±2.9	1.9 ±2.9	1.9 ±2.9
Transitional Chinese medicine	2.0 ±3.2	2.0 ±3.2	2.0 ±3.2	2.3 ±3.3	2.3 ±3.3	2.3 ±3.3
Dentistry	1.5 ±2.3	1.5 ±2.3	1.5 ±2.3	1.6 ±2.4	1.6 ±2.4	1.6 ±2.4
**Number of emergency medicine**	0.4 ±1.0	0.4 ±1.0	0.4 ±1.1	0.7 ±1.5	0.7 ±1.5	0.7 ±1.5
**Inpatient treatment**						
Psychiatry	6,862 (1.0)	5,489 (1.0)	1,373 (1.0)	31,640 (4.3)	25,476 (4.3)	6,164 (4.1)
Neurology	9,914 (1.4)	7,916 (1.4)	1,998 (1.4)	10,484 (1.4)	8,463 (1.4)	2,021 (1.4)
Internal medicine	44,749 (6.3)	35,799 (6.3)	8,950 (6.3)	52,314 (7.1)	41,900 (7.1)	10,414 (7.0)
Surgery	26,001 (3.6)	20,777 (3.6)	5,224 (3.7)	32,555 (4.4)	25,948 (4.4)	6,607 (4.4)
Obstetrics and gynecology	16,287 (2.3)	13,014 (2.3)	3,273 (2.3)	14,951 (2.0)	11,901 (2.0)	3,050 (2.1)
Orthopedics	13,460 (1.9)	10,820 (1.9)	2,640 (1.8)	18,187 (2.5)	14,740 (2.5)	3,447 (2.3)
Urologist	5,372 (0.8)	4,322 (0.8)	1,050 (0.7)	6,000 (0.8)	4,845 (0.8)	1,155 (0.8)
Other specialties	17,825 (2.5)	14,260 (2.5)	3,565 (2.5)	19,332 (2.6)	15,507 (2.6)	3,825 (2.6)
**Medication use, days**						
Stomatological Preparations (ATC code:A01)	0.6 ±4.3	0.6 ±4.3	0.6 ±4.3	0.8 ±5.1	0.8 ±5.1	0.7 ±4.9
Antacids (ATC code:A02A)	13.5 ±24.5	13.5 ±24.5	13.6 ±24.6	15.8 ±26.7	15.9 ±26.8	15.6 ±26.5
Drugs for Peptic Ulcer and Gastro-esophageal Reflux Disease (ATC code:A02B)	6.0 ±16.6	6.0 ±16.6	6.1 ±16.6	7.8 ±19.3	7.8 ±19.3	7.7 ±19.1
Drugs for Functional Gastrointestinal Disorders (ATC code:A03A)	7.6 ±16.2	7.6 ±16.2	7.7 ±16.2	9.8 ±19.4	9.7 ±19.4	9.8 ±19.3
Belladonna and Derivatives, Plain (ATC code:A03B)	1.3 ±4.9	1.3 ±4.9	1.3 ±4.8	1.6 ±5.7	1.6 ±5.8	1.5 ±5.5
Propulsives (ATC code:A03F)	6.4 ±16.3	6.4 ±16.3	6.4 ±16.2	8.7 ±19.1	8.7 ±19.1	8.7 ±19.2
Antiemetics and Antinauseants (ATC code:A04)	0.7 ±3.6	0.7 ±3.6	0.7 ±3.6	0.8 ±3.9	0.8 ±3.8	0.8 ±4.0

Bile and Liver Therapy (ATC code:A05)	1.8 ±10.0	1.8 ±10.0	1.8 ±10.1	2.0 ±10.8	2.0 ±10.9	2.0 ±10.6
Drugs for Constipation (ATC code:A06)	3.9 ±15.1	3.9 ±15.1	3.9 ±14.9	7.7 ±22.3	7.8 ±22.4	7.5 ±21.9
Antidiarrheals, Intestinal Antiinflammatory/Antiinfective Agents (ATC code:A07)	1.9 ±7.6	1.9 ±7.6	1.9 ±7.5	2.6 ±9.4	2.6 ±9.6	2.4 ±8.7
Digestives, Incl. Enzymes (ATC code:A09)	1.2 ±6.6	1.2 ±6.6	1.2 ±6.6	1.6 ±7.7	1.6 ±7.7	1.6 ±7.7
Drugs Used In Diabetes (ATC code:A10)	4.1 ±18.7	4.1 ±18.7	4.2 ±18.9	3.8 ±17.9	3.8 ±17.8	3.9 ±18.4
Vitamins (ATC code:A11)	2.6 ±10.7	2.6 ±10.7	2.6 ±10.9	3.3 ±12.3	3.3 ±12.2	3.2 ±12.3
Mineral Supplements (ATC code:A12)	1.0 ±6.4	1.0 ±6.4	1.0 ±6.6	1.0 ±6.2	1.0 ±6.1	1.0 ±6.3
Antithrombotic Agents (ATC code:B01)	5.0 ±18.6	4.9 ±18.5	5.0 ±18.9	4.3 ±16.9	4.2 ±16.8	4.4 ±17.3
Antihemorrhagics (ATC code:B02)	0.9 ±4.3	0.9 ±4.3	0.9 ±4.2	0.9 ±4.1	0.9 ±4.1	0.9 ±4.1
Antianemic Preparations (ATC code:B03)	1.9 ±11.0	1.9 ±11.0	1.9 ±11.1	2.1 ±11.4	2.1 ±11.4	2.1 ±11.2
Blood Substitutes and Perfusion Solutions (ATC code:B05)	2.8 ±9.9	2.8 ±9.9	2.9 ±10.0	3.1 ±9.8	3.1 ±9.8	3.1 ±9.9
Other Hematological Agents (ATC code:B06)	1.4 ±4.8	1.4 ±4.8	1.4 ±4.8	1.6 ±5.3	1.6 ±5.2	1.6 ±5.6
Cardiac Therapy (ATC code:C01)	2.4 ±12.6	2.4 ±12.5	2.5 ±13.0	2.1 ±11.5	2.1 ±11.5	2.2 ±11.6
Antihypertensives (ATC code:C02)	1.1 ±7.9	1.1 ±7.9	1.1 ±8.0	1.0 ±7.4	1.0 ±7.5	0.9 ±7.3
Diuretics (ATC code:C03)	2.8 ±13.3	2.8 ±13.2	2.9 ±13.5	2.5 ±12.4	2.6 ±12.4	2.5 ±12.5
Peripheral Vasodilators (ATC code:C04)	2.3 ±9.9	2.3 ±9.9	2.4 ±10.1	2.7 ±10.6	2.7 ±10.6	2.7 ±10.5
Vasoprotectives (ATC code:C05)	0.8 ±5.1	0.8 ±5.1	0.8 ±5.1	1.0 ±5.8	1.0 ±5.8	1.0 ±5.7
Beta Blocking Agents (ATC code:C07)	8.7 ±22.3	8.7 ±22.2	8.8 ±22.4	17.4 ±32.0	17.3 ±31.9	17.7 ±32.3
Calcium Channel Blockers (ATC code:C08)	6.8 ±21.5	6.8 ±21.4	6.9 ±21.7	6.8 ±21.8	6.8 ±21.6	7.1 ±22.2
Agents Acting On The Renin-Angiotensin System (ATC code:C09)	5.4 ±19.1	5.4 ±19.1	5.4 ±19.2	5.0 ±18.3	4.9 ±18.3	5.1 ±18.5
Lipid Modifying Agents (ATC code:C10)	3.5 ±14.3	3.5 ±14.2	3.6 ±14.5	3.8 ±14.7	3.8 ±14.7	3.9 ±14.9
Antifungals for Dermatological Use (ATC code:D01)	1.3 ±5.5	1.3 ±5.5	1.3 ±5.4	1.5 ±6.1	1.5 ±6.1	1.5 ±6.3
Emollients and Protectives (ATC code:D02)	0.4 ±3.3	0.4 ±3.2	0.4 ±3.3	0.4 ±3.3	0.4 ±3.3	0.4 ±3.3
Antipsoriatics (ATC code:D05)	0.2 ±2.4	0.2 ±2.4	0.2 ±2.3	0.2 ±2.8	0.2 ±2.8	0.2 ±2.8
Antibiotics and Chemotherapeutics for Dermatological Use (ATC code:D06)	0.9 ±4.6	0.9 ±4.6	0.9 ±4.5	1.2 ±5.2	1.2 ±5.2	1.1 ±5.1
Corticosteroids, Dermatological Preparations (ATC code:D07)	2.8 ±8.1	2.8 ±8.1	2.8 ±7.9	3.3 ±9.1	3.3 ±9.0	3.4 ±9.3

Anti-Acne Preparations (ATC code:D10)	0.5 ±2.9	0.5 ±2.9	0.5 ±2.8	0.6 ±3.5	0.6 ±3.5	0.6 ±3.4
Gynecological Antiinfectives and Antiseptics (ATC code:G01)	1.1 ±4.2	1.1 ±4.2	1.1 ±4.3	1.3 ±4.9	1.3 ±4.9	1.3 ±4.9
Sex Hormones and Modulators Of The Genital System (ATC code:G03)	2.9 ±10.9	2.9 ±10.9	2.9 ±11.0	3.5 ±12.1	3.5 ±12.1	3.5 ±12.1
Urologicals (ATC code:G04)	2.1 ±10.0	2.1 ±10.0	2.1 ±10.0	2.5 ±10.7	2.5 ±10.7	2.5 ±10.6
Corticosteroids for Systemic Use (ATC code:H02)	2.7 ±9.8	2.7 ±9.8	2.7 ±9.8	2.9 ±10.2	2.9 ±10.3	2.9 ±10.0
Thyroid Therapy (ATC code:H03)	0.8 ±7.2	0.8 ±7.2	0.8 ±7.3	1.0 ±8.1	1.0 ±7.9	1.1 ±8.9
Antibacterials for Systemic Use (ATC code:J01)	5.5 ±10.5	5.5 ±10.5	5.5 ±10.5	5.9 ±11.1	5.9 ±11.1	5.9 ±11.1
Antimycotics for Systemic Use (ATC code:J02)	0.3 ±2.5	0.3 ±2.5	0.3 ±2.4	0.2 ±2.4	0.2 ±2.4	0.2 ±2.3
Antivirals for Systemic Use (ATC code:J05)	0.7 ±4.3	0.7 ±4.4	0.7 ±4.3	0.8 ±4.9	0.8 ±4.8	0.8 ±5.4
Vaccines (ATC code:J07)	0.3 ±1.8	0.3 ±1.8	0.3 ±1.8	0.4 ±1.9	0.4 ±1.9	0.4 ±2.0
Immunostimulants (ATC code:L03)	0.2 ±3.2	0.2 ±3.3	0.2 ±3.2	0.3 ±3.5	0.3 ±3.4	0.3 ±3.5
Antiinflammatory and Antirheumatic Products (ATC code:M01)	11.7 ±20.0	11.6 ±20.0	11.7 ±20.0	14.5 ±23.9	14.6 ±24.0	14.4 ±23.7
Topical Products for Joint and Muscular Pain (ATC code:M02)	2.2 ±9.1	2.2 ±9.1	2.2 ±9.0	3.0 ±11.0	3.0 ±11.0	2.9 ±11.1
Muscle Relaxants (ATC code:M03)	5.2 ±13.0	5.2 ±13.0	5.2 ±13.0	7.3 ±16.6	7.3 ±16.7	7.2 ±16.3
Antigout Preparations (ATC code:M04)	1.4 ±9.1	1.4 ±9.1	1.4 ±9.4	1.2 ±8.4	1.2 ±8.4	1.2 ±8.5
Opioids (ATC code:N02A)	1.4 ±8.0	1.4 ±8.1	1.4 ±7.8	2.0 ±10.2	2.0 ±10.3	2.0 ±10.0
Other Analgesics and Antipyretics (ATC code:N02B)	8.7 ±15.3	8.7 ±15.3	8.7 ±15.4	12.1 ±20.6	12.1 ±20.6	12.0 ±20.5
Antimigraine Preparations (ATC code:N02C)	0.5 ±4.2	0.5 ±4.2	0.5 ±4.2	1.0 ±7.1	1.1 ±7.2	1.0 ±6.7
Antiepileptics (ATC code:N03A)	2.9 ±12.5	2.9 ±12.5	3.0 ±12.6	15.7 ±30.3	15.8 ±30.5	15.5 ±29.9
Anticholinergic Agents (ATC code:N04A)	0.2 ±2.9	0.2 ±3.0	0.2 ±2.8	1.1 ±8.4	1.1 ±8.6	1.1 ±7.9
Dopaminergic Agents (ATC code:N04B)	0.2 ±2.4	0.2 ±2.4	0.2 ±2.6	0.4 ±4.4	0.4 ±4.4	0.4 ±4.3
Antipsychotics (First-Generation Antipsychotics Only) (ATC code:N05A)	0.4 ±3.9	0.4 ±3.9	0.4 ±3.9	2.3 ±12.2	2.3 ±12.3	2.3 ±11.8
Anxiolytics (ATC code:N05B)	17.9 ±29.9	17.9 ±29.8	18.1 ±30.1	46.7 ±50.7	46.6 ±50.6	47.1 ±51.3
Hypnotics and Sedatives (ATC code:N05C)	15.1 ±32.1	15.1 ±32.0	15.3 ±32.6	54.1 ±64.9	54.0 ±64.9	54.5 ±64.9
Psychostimulants, Agents Used for Adhd and Nootropics (ATC code:N06B)	0.6 ±5.4	0.6 ±5.4	0.6 ±5.5	1.0 ±7.1	1.0 ±7.1	1.0 ±6.9
Parasympathomimetics (ATC code:N07A)	0.3 ±3.5	0.2 ±3.5	0.3 ±3.6	0.4 ±4.4	0.4 ±4.3	0.4 ±4.6
Antivertigo Preparations (ATC code:N07C)	4.1 ±13.2	4.0 ±13.2	4.1 ±13.5	5.6 ±16.1	5.6 ±16.1	5.6 ±16.1
Nasal Preparations (ATC code:R01)	3.3 ±9.8	3.3 ±9.8	3.3 ±9.8	4.0 ±11.5	4.0 ±11.4	4.0 ±11.8

Drugs for Obstructive Airway Diseases (ATC code:R03)	5.2 ±19.8	5.2 ±19.8	5.2 ±19.7	5.7 ±20.8	5.7 ±20.9	5.8 ±20.4
Cough and Cold Preparations (ATC code:R05)	12.2 ±24.9	12.2 ±24.9	12.4 ±25.2	14.1 ±27.8	14.2 ±27.8	14.0 ±27.5
Antihistamines for Systemic Use (ATC code:R06)	7.4 ±14.8	7.4 ±14.8	7.5 ±15.0	9.4 ±17.9	9.3 ±17.9	9.4 ±17.9
Ophthalmologicals (ATC code:S01)	3.5 ±10.0	3.5 ±10.0	3.5 ±10.0	3.8 ±10.8	3.8 ±10.8	3.9 ±11.1
**History of previous antidepressant regimen in the past year**						
**Psychotropic agent use in the past year (days)**						
Amitriptyline				1.5 ±9.6	1.5 ±9.6	1.5 ±9.8
Bupropion				2.8 ±11.9	2.8 ±11.7	2.9 ±12.4
Citalopram				4.7 ±16.2	4.6 ±15.8	4.9 ±17.4
Doxepin				1.0 ±7.4	1.0 ±7.4	0.9 ±7.4
Duloxetine				2.5 ±11.9	2.5 ±11.9	2.4 ±11.9
Escitalopram				5.3 ±17.1	5.3 ±17.1	5.4 ±17.1
Fluoxetine				8.7 ±21.5	8.7 ±21.5	8.6 ±21.3
Fluvoxamine				2.0 ±10.8	2.1 ±10.9	2.0 ±10.4
Imipramine				2.2 ±10.3	2.3 ±10.4	2.2 ±10.0
Milnacipran				0.5 ±5.2	0.5 ±5.2	0.6 ±5.2
Mirtazapine				7.0 ±21.3	7.0 ±21.4	6.9 ±21.1
Moclobemide				1.0 ±7.0	1.0 ±7.0	1.0 ±7.1
Paroxetine				7.2 ±20.1	7.2 ±20.1	7.1 ±19.9
Sertraline				7.8 ±20.0	7.7 ±20.0	7.9 ±20.3
Trazodone				14.7 ±31.6	14.6 ±31.6	14.8 ±31.7
Venlafaxine				6.4 ±19.3	6.3 ±19.3	6.6 ±19.5
Lamotrigine				0.3 ±4.7	0.3 ±4.7	0.3 ±4.5
Lithium				0.3 ±4.4	0.4 ±4.5	0.3 ±4.1
Valproic Acid				1.4 ±9.0	1.4 ±9.1	1.3 ±8.5
Amisulpride				0.2 ±3.5	0.3 ±3.5	0.2 ±3.4
Aripiprazole				0.4 ±4.8	0.4 ±4.8	0.4 ±4.5
Olanzapine				0.4 ±4.9	0.4 ±4.9	0.4 ±5.0
Quetiapine				3.6 ±16.2	3.6 ±16.3	3.5 ±16.1
Risperidone				0.5 ±5.4	0.5 ±5.5	0.5 ±5.3
Zotepine				0.3 ±4.0	0.3 ±4.0	0.3 ±3.9
**Gap between the last and the current treatment**				99.9 ±146.1	100.0 ±146.1	99.6 ±146.1
**Number of failed treatment episodes in the past year**						
1				274,551 (37.1)	219,373 (37.1)	55,178 (37.1)
2				144,453 (19.5)	115,366 (19.5)	29,087 (19.6)

3-4	140,561<br>(19.0)	112,271<br>(19.0)	28,290<br>(19.0)
≥ 5	180,415<br>(24.4)	144,414<br>(24.4)	36,001<br>(24.2)

Supplementary Table S2. Study design for target trials

Component	Target Trial	Emulated trial using real world data
Eligibility	Patients with incident depressive disorder who initiated or changed antidepressant regimen between the year 2003 and 2012	Patients with incident depression was defined as no previous diagnosis in 2-year washout period (2001-2002)
Treatment assignment	**Treatment groups**<br>Treatment based on the recommendations by predictive model<br>**Comparison groups**<br>1. Treatment as usual<br>2. Treatment was randomly-selected by the prescription proportion of the observed treatment<br>3. Treatment was randomly-selected by the recommendation proportion<br>**Patients are randomly assigned to either strategy**	**Four replicated sample. Restriction to the patients treated with the assigned group**<br>Randomization is emulated via adjustment for baseline covariates
Outcome	Composite treatment failure, either one of the following conditions: (1) psychiatric hospitalization, (2) self-harm hospitalization, (3) emergency visits for psychiatric problem, or (4) treatment change	Same
Follow-up	Follow-up starts at treatment assignment with an interval of one month and ends at treatment failure, loss to follow-up, or 1 year after baseline, whichever comes first.	Same
Statistical analysis	ITT-analysis, as-treated analysis: comparison of 1-year risk of treatment failure between groups receiving each treatment strategies with adjustment of baseline and post-baseline covariates when adjusting for discontinuation or loss of follow-up.	Same

Supplementary Table S3. Comparisons of the performance of the prediction models using Super Learner ensemble, logistic regression, random forest, and support vector machine

	The initial treatment, AUC (95% CI)	The next-step treatment, AUC (95% CI)
Super Learner ensemble	0.627 (0.623, 0.630)	0.751 (0.747, 0.756)
Logistic regression	0.614 (0.610, 0.617)	0.677 (0.670, 0.683)
Random forest	0.602 (0.599, 0.606)	0.732 (0.726, 0.738)
Support vector machine	0.573 (0.570, 0.577)	0.657 (0.650, 0.664)

* the performance of prediction models is measured using the area under the curve (AUC) of receiver operator characteristic analyses

Supplementary Table S4. Distribution of observed and recommended treatment among test datasets.

**Initial treatment episode (n=143,042)**	**Observed (%)**	**Recommended (%)**
Amitriptyline	0.4	0.5
Bupropion	3.4	2.7
Citalopram	8.8	0.8
Doxepin	0.3	1.1
Duloxetine	2.7	3.6
Escitalopram	10.0	8.0
Fluoxetine	22.2	46.9
Fluvoxamine	2.7	0.3
Imipramine	0.8	2.2
Milnacipran	0.5	2.2
Mirtazapine	7.3	3.0
Moclobemide	2.6	16.8
Paroxetine	12.6	2.6
Sertraline	18.1	8.0
Trazodone	0.6	1.2
Venlafaxine	7.0	0.2
**Next-step treatment episodes (n=148,556)**	**Observed (%)[a]**	**Recommended (%)[a]**
**Switching (n=135,174)**		
Amitriptyline	0.5	9.0
Bupropion	5.3	8.6
Citalopram	8.2	2.0
Doxepin	0.5	5.6
Duloxetine	4.5	14.9
Escitalopram	11.4	1.6
Fluoxetine	14.4	1.4
Fluvoxamine	3.1	1.5
Imipramine	0.8	3.9
Milnacipran	0.9	6.8
Mirtazapine	11.2	3.7
Moclobemide	2.0	10.0
Paroxetine	11.2	2.0
Sertraline	14.5	18.0
Trazodone	1.1	6.0
Venlafaxine	10.2	5.0
**Combinations (n=4,265)**		
Amitriptyline	3.0	4.4
Bupropion	19.8	3.7
Citalopram	2.9	2.3
Doxepin	2.5	12.9
Duloxetine	5.3	5.5
Escitalopram	6.5	28.1
Fluoxetine	9.8	8.9
Fluvoxamine	1.5	1.2

Imipramine	3.6	1.3
Milnacipran	1.0	0.9
Mirtazapine	15.1	2.2
Moclobemide	1.1	7.4
Paroxetine	5.9	3.4
Sertraline	6.1	9.8
Trazodone	6.3	1.4
Venlafaxine	9.6	6.6
**Augmentations (n=9,117)**		
Amisulpride	4.2	8.6
Aripiprazole	9.8	7.4
Olanzapine	5.9	18.2
Quetiapine	42.2	1.9
Risperidone	7.3	0.5
Zotepine	3.5	20.3
Lamotrigine	3.9	11.4
Lithium	5.0	27.9
Valproic acid	18.3	3.9

* the percentage was calculated by each strategy for the next-step treatment

Supplementary Table S5. The baseline characteristics of these four groups for the initial treatment

	Model recommend (n=23,965)	As usual (n=143,042)	Randomly selected by prescription proportion (n=18,485)	Randomly selected by recommended proportion (n=19,924)
Age group, n (%)				
18-29	5,709 (23.8)	38,766 (27.1) *	5,146 (27.8)*	5,669 (28.5)*
30-39	4,913 (20.5)	28,796 (20.1) *	3,759 (20.3)*	3,995 (20.1)*
40-49	4,854 (20.3)	28,578 (20.0) *	3,665 (19.8)*	3,957 (19.9)*
50-59	4,619 (19.3)	24,648 (17.2) *	3,089 (16.7)*	3,255 (16.3)*
≥60	3,870 (16.1)	22,254 (15.6) *	2,826 (15.3)*	3,048 (15.3)*
Sex, female	16,616 (69.3)	92,468 (64.6)*	12,203 (66.0)*	13,212 (66.3)*
**Type of depression**				
Major depressive disorder	6,450 (26.9)	40,612 (28.4)*	5,325 (28.8)*	5,696 (28.6)*
Dysthymic disorder	13,974 (58.3)	78,579 (54.9)*	10,083 (54.5)*	10,862 (54.5)*
Depressive disorder, NOS	3,541 (14.8)	23,851 (16.7)*	3,077 (16.6)*	3,366 (16.9)*
**Duration of illness**	0.2 ±0.9	0.2 ±1.0*	0.2 ±0.9	0.2 ±0.8*
**Comorbidity**				
Diabetes mellitus (ICD-9-CM: 250)	2,173 (9.1)	12,889 (9.0)	1,637 (8.9)	1,776 (8.9)
Dyslipidemia (ICD-9-CM: 272)	3,324 (13.9)	18,529 (13.0)*	2,379 (12.9)*	2,513 (12.6)*
Asthma (ICD-9-CM: 493)	1,170 (4.9)	6,482 (4.5)*	813 (4.4)*	935 (4.7)
Cancer (ICD-9-CM: 140-208, 273.0, 273.3, and V10)	811 (3.4)	5,389 (3.8)*	668 (3.6)	651 (3.3)
Headache (ICD-9-CM: 307.81, 346, 784.0)	7,874 (32.9)	43,554 (30.4)*	5,716 (30.9)*	6,240 (31.3)*
Hemiplegia (ICD-9-CM: 342, 344)	263 (1.1)	1,606 (1.1)	239 (1.3)	234 (1.2)
Peripheral neurological disorder (ICD-9-CM: 351-357)	1,719 (7.2)	10,140 (7.1)	1,294 (7.0)	1,373 (6.9)
Cerebrovascular disease (ICD-9-CM: 362.34, 430-438, 781.4, 784.3, 997.0)	1,357 (5.7)	7,377 (5.2)*	991 (5.4)	1,037 (5.2)*
Hypertension (ICD-9-CM: 401-405)	5,535 (23.1)	28,638 (20.0)*	3,708 (20.1)*	4,058 (20.4)*
Congestive heart failure (ICD-9-CM: 402.01, 402.11, 302.91, 425, 428, 429.3)	646 (2.7)	3,231 (2.3)*	412 (2.2)*	434 (2.2)*
Chronic kidney disease (ICD-9-CM: 403.x1, 404.x2, 585, 586, V42.0, V45.1, V56.0, V56.8)	295 (1.2)	1,872 (1.3)	230 (1.2)	225 (1.1)
Coronary heart disease (ICD-9-CM: 410-414)	2,266 (9.5)	12,345 (8.6)*	1,593 (8.6)*	1,705 (8.6)*
Peripheral vascular disease (ICD-9-CM: 440, 441.2, 441.4, 441.7, 441.9, 443.1- 443.9, 447.1, 557, 785.4)	406 (1.7)	2,064 (1.4)*	255 (1.4)*	290 (1.5)*
Chronic pulmonary disease (ICD-9-CM: 491, 492, 494, 495, 496)	1,280 (5.3)	6,949 (4.9)*	859 (4.6)*	917 (4.6)*
Peptic ulcer disease (ICD-9-CM: 531 – 534)	4,271 (17.8)	25,334 (17.7)	3,378 (18.3)	3,547 (17.8)
Functional gastrointestinal disorders (ICD-9-CM: 536, 564, 306.4)	8,710 (36.3)	48,380 (33.8)*	6,395 (34.6)*	6,905 (34.7)*
Chronic liver disease (ICD-9-CM: 571.2-571.9; 456.0-456.2)	576 (2.4)	3,627 (2.5)	476 (2.6)	494 (2.5)

Rheumatological disease (ICD-9-CM: 710.0, 710.1, 710.4, 714.0, 714.1, 714.2, 714.81, 725)	355 (1.5)	1,967 (1.4)	238 (1.3)	289 (1.5)
Fibromyalgia and osteoarthritis (ICD-9-CM: 715, 729.1)	6,785 (28.3)	43,270 (30.2)*	5,650 (30.6)*	6,032 (30.3)*
Back pain (ICD-9-CM: 721, 722, 723, 724, 739.3,739.4, 846, 847.2)	7,684 (32.1)	44,280 (31.0)*	5,805 (31.4)	6,180 (31.0)*
Alcohol use disorder (ICD-9-CM: 291, 303.9, 305.0, 357.5, 425.5, 535.3, 571.0-571.3, V11.3)	574 (2.4)	4,067 (2.8)*	505 (2.7)*	443 (2.2)
Substance use disorder (ICD-9-CM: 292, 304, 305.2-305.9)	139 (0.6)	1,380 (1.0)*	155 (0.8)*	145 (0.7)
Anxiety state, unspecified (ICD-9-CM: 300 without 4th or 5th digit)	300 (1.3)	1,669 (1.2)	195 (1.1)	219 (1.1)
Panic disorder (ICD-9-CM: 300.01)	895 (3.7)	6,363 (4.4)*	837 (4.5)*	758 (3.8)
Generalized anxiety disorder (ICD-9-CM: 300.02)	2,032 (8.5)	11,984 (8.4)	1,544 (8.4)	1,384 (6.9)*
Phobic disorders (ICD-9-CM: 300.2)	155 (0.6)	1,110 (0.8)*	129 (0.7)	111 (0.6)
Neurasthenia (ICD-9-CM: 300.5)	229 (1.0)	1,133 (0.8)*	171 (0.9)	191 (1.0)
Personality disorders (ICD-9-CM: 301)	143 (0.6)	1,075 (0.8)*	122 (0.7)	146 (0.7)
Adjustment disorders (ICD-9-CM: 309)	1,375 (5.7)	10,054 (7.0)*	1,259 (6.8)*	1,223 (6.1)
Obsessive-compulsive disorders (ICD-9-CM:300.3)	443 (1.8)	1,850 (1.3)*	248 (1.3)*	299 (1.5)*
Somatoform disorders (ICD-9-CM:300.8)	264 (1.1)	1,955 (1.4)*	229 (1.2)	239 (1.2)
Other anxiety disorders (ICD-9-CM:300.1, 300.6, 300.7, 300.9)	2,012 (8.4)	10,275 (7.2)*	1,301 (7.0)*	1,416 (7.1)*
Physiological malfunction arising from mental factors (ICD-9-CM:306)	1,203 (5.0)	6,085 (4.3)*	822 (4.4)*	906 (4.5)*
Sleep disorders (ICD-9-CM:307.4, 780.5)	11,510 (48.0)	66,789 (46.7)*	8,635 (46.7)*	8,726 (43.8)*
Eating disorders (ICD-9-CM: 307.1, 307.5)	257 (1.1)	793 (0.6)*	136 (0.7)*	215 (1.1)
**Number of outpatient visits**				
Psychiatry	0.7 ±2.0	0.8 ±2.1*	0.7 ±2.0	0.6 ±1.9*
Family medicine	3.8 ±3.8	3.2 ±3.6*	3.3 ±3.6*	3.4 ±3.7*
Internal medicine	2.8 ±3.5	2.8 ±3.5*	2.8 ±3.5*	2.7 ±3.5
Dermatology	0.7 ±1.6	0.8 ±1.8*	0.8 ±1.8*	0.8 ±1.8*
Neurology	0.6 ±1.7	0.6 ±1.7	0.6 ±1.7	0.6 ±1.7
Obstetrics and gynecology	1.4 ±2.6	1.3 ±2.6*	1.4 ±2.6	1.4 ±2.6
Ophthalmology	1.0 ±2.0	0.9 ±2.0*	0.9 ±2.0*	0.9 ±1.9*
Orthopedics	0.7 ±1.8	0.7 ±1.8	0.7 ±1.8	0.7 ±1.8
Otolaryngology	1.4 ±2.5	1.6 ±2.6*	1.6 ±2.6*	1.5 ±2.6*
Rehabilitation	0.3 ±1.4	0.4 ±1.5*	0.4 ±1.5*	0.4 ±1.5
Surgery	0.6 ±1.6	0.6 ±1.6*	0.6 ±1.6*	0.6 ±1.6*
Other specialties	1.4 ±2.6	1.6 ±2.7*	1.5 ±2.6*	1.5 ±2.6*
Transitional Chinese medicine	2.1 ±3.2	2.0 ±3.2*	2.1 ±3.2	2.1 ±3.2*
Dentistry	1.4 ±2.3	1.5 ±2.3	1.5 ±2.3*	1.4 ±2.2
**Number of Emergency medicine**	0.3 ±1.0	0.4 ±1.1*	0.4 ±1.0*	0.4 ±1.0*
**Inpatient treatment, n (%)**				

Psychiatry	119 (0.5)	1,373 (1.0)*	160 (0.9)*	140 (0.7)*
Neurology	319 (1.3)	1,998 (1.4)	256 (1.4)	279 (1.4)
Internal medicine	1,322 (5.5)	8,950 (6.3)*	1,139 (6.2)*	1,066 (5.4)
Surgery	698 (2.9)	5,224 (3.7)*	668 (3.6)*	654 (3.3)*
Obstetrics and gynecology	525 (2.2)	3,273 (2.3)	431 (2.3)	475 (2.4)
Orthopedics	393 (1.6)	2,640 (1.8)*	327 (1.8)	345 (1.7)
Urologist	142 (0.6)	1,050 (0.7)*	126 (0.7)	136 (0.7)
Other specialties	510 (2.1)	3,565 (2.5)*	438 (2.4)	464 (2.3)
**Medication use, days**				
Stomatological Preparations (ATC code:A01)	4.3 ±28.6	5.0 ±31.1*	4.6 ±29.1	4.3 ±28.5
Antacids (ATC code:A02A)	43.2 ±71.3	40.7 ±69.6*	41.4 ±69.3*	42.4 ±70.3
Drugs for Peptic Ulcer and Gastro-esophageal Reflux Disease (ATC code:A02B)	20.0 ±53.7	20.8 ±55.2	20.9 ±55.8	20.0 ±53.7
Drugs for Functional Gastrointestinal Disorders (ATC code:A03A)	22.2 ±45.0	21.0 ±43.9*	21.5 ±44.3	21.2 ±44.1*
Belladonna and Derivatives, Plain (ATC code:A03B)	6.2 ±23.8	6.2 ±23.1	6.4 ±23.5	6.2 ±22.6
Propulsives (ATC code:A03F)	19.4 ±47.7	20.2 ±49.8*	20.1 ±49.5	19.3 ±47.8
Antiemetics and Antinauseants (ATC code:A04)	5.5 ±27.2	5.5 ±26.6	5.2 ±24.5	5.2 ±23.9
Bile and Liver Therapy (ATC code:A05)	6.3 ±33.2	6.2 ±33.6	6.9 ±35.8	6.4 ±34.5
Drugs for Constipation (ATC code:A06)	15.5 ±55.2	16.5 ±57.2*	16.3 ±56.7	15.1 ±53.7
Antidiarrheals, Intestinal Antiinflammatory/Antiinfective Agents (ATC code:A07)	11.8 ±41.8	11.1 ±39.4*	10.8 ±37.6*	10.7 ±38.1*
Digestives, Incl. Enzymes (ATC code:A09)	7.3 ±35.9	6.9 ±34.5	7.0 ±34.3	6.8 ±33.5
Drugs Used In Diabetes (ATC code:A10)	9.9 ±43.6	10.4 ±45.7	10.3 ±45.7	10.4 ±45.8
Vitamins (ATC code:A11)	10.5 ±39.2	10.9 ±41.4	11.1 ±41.0	10.8 ±41.3
Mineral Supplements (ATC code:A12)	8.8 ±49.3	9.0 ±49.5	8.1 ±47.0	8.2 ±47.2
Antithrombotic Agents (ATC code:B01)	19.9 ±64.1	17.3 ±60.1*	17.3 ±60.2*	17.5 ±60.5*
Antihemorrhagics (ATC code:B02)	11.3 ±43.8	11.5 ±43.7	11.8 ±43.7	11.2 ±43.0
Antianemic Preparations (ATC code:B03)	8.6 ±42.1	8.8 ±43.3	9.4 ±44.9*	8.9 ±42.9
Blood Substitutes and Perfusion Solutions (ATC code:B05)	13.2 ±40.9	14.2 ±42.4*	13.5 ±40.1	12.9 ±39.3
Other Hematological Agents (ATC code:B06)	15.7 ±45.8	15.5 ±45.9	15.7 ±45.4	15.5 ±44.6
Cardiac Therapy (ATC code:C01)	10.8 ±50.6	10.9 ±50.8	10.2 ±48.7	10.2 ±48.8
Antihypertensives (ATC code:C02)	3.9 ±25.4	3.3 ±24.0*	3.4 ±24.4	3.7 ±25.2
Diuretics (ATC code:C03)	13.4 ±53.4	12.0 ±51.6*	12.0 ±51.6*	12.5 ±52.7
Peripheral Vasodilators (ATC code:C04)	10.6 ±41.2	9.8 ±39.5*	9.9 ±40.1	9.6 ±38.9*
Vasoprotectives (ATC code:C05)	7.7 ±42.3	9.2 ±47.0*	9.3 ±47.6*	9.1 ±46.8*
Beta Blocking Agents (ATC code:C07)	25.4 ±61.0	23.9 ±59.0*	24.2 ±59.6*	23.6 ±57.9*

Calcium Channel Blockers (ATC code:C08)	22.2 ±62.8	19.9 ±60.0*	19.7 ±60.0*	20.1 ±60.9*
Agents Acting On The Renin-Angiotensin System (ATC code:C09)	16.8 ±54.8	15.4 ±52.9*	15.3 ±53.4*	15.2 ±52.7*
Lipid Modifying Agents (ATC code:C10)	11.2 ±43.4	11.1 ±43.5	11.1 ±43.6	10.5 ±42.9
Antifungals for Dermatological Use (ATC code:D01)	7.4 ±30.1	7.8 ±30.8*	7.9 ±30.5	8.2 ±31.9*
Emollients and Protectives (ATC code:D02)	7.7 ±44.3	8.6 ±47.4*	8.5 ±47.3	8.6 ±47.4*
Antipsoriatics (ATC code:D05)	2.6 ±29.4	3.8 ±35.6*	3.8 ±35.9*	3.3 ±33.2*
Antibiotics and Chemotherapeutics for Dermatological Use (ATC code:D06)	3.7 ±19.1	4.6 ±22.0*	4.3 ±20.0*	4.1 ±20.6*
Corticosteroids, Dermatological Preparations (ATC code:D07)	13.5 ±37.0	16.4 ±43.2*	16.8 ±43.9*	16.3 ±42.1*
Anti-Acne Preparations (ATC code:D10)	9.9 ±42.9	11.9 ±48.2*	11.9 ±48.2*	12.2 ±48.9*
Gynecological Antiinfectives and Antiseptics (ATC code:G01)	23.6 ±66.7	22.5 ±66.8*	23.9 ±68.2	24.0 ±68.4
Sex Hormones and Modulators Of The Genital System (ATC code:G03)	16.4 ±54.9	15.2 ±52.7*	15.5 ±53.2	15.8 ±53.7
Urologicals (ATC code:G04)	9.6 ±42.5	10.5 ±44.7*	10.5 ±44.0*	9.8 ±43.3
Corticosteroids for Systemic Use (ATC code:H02)	8.9 ±31.0	8.8 ±30.4	8.4 ±28.8	8.5 ±29.9
Thyroid Therapy (ATC code:H03)	3.8 ±30.9	3.7 ±30.7	3.6 ±30.1	3.6 ±30.3
Antibacterials for Systemic Use (ATC code:J01)	21.8 ±40.2	22.7 ±41.4*	22.5 ±40.3*	22.2 ±39.3
Antimycotics for Systemic Use (ATC code:J02)	3.0 ±23.9	2.9 ±23.0	3.0 ±22.9	3.0 ±23.1
Antivirals for Systemic Use (ATC code:J05)	11.6 ±35.4	13.1 ±41.9*	13.1 ±41.3*	13.0 ±40.7*
Vaccines (ATC code:J07)	5.0 ±25.0	6.0 ±28.4*	6.1 ±29.0*	5.8 ±27.6*
Immunostimulants (ATC code:L03)	2.1 ±24.1	2.4 ±25.9	2.6 ±27.2	2.2 ±24.8
Antiinflammatory and Antirheumatic Products (ATC code:M01)	31.2 ±52.4	30.7 ±51.4	31.1 ±51.2	30.9 ±51.4
Topical Products for Joint and Muscular Pain (ATC code:M02)	11.9 ±46.5	13.1 ±49.0*	13.1 ±48.7*	12.8 ±48.0*
Muscle Relaxants (ATC code:M03)	15.9 ±39.0	16.5 ±40.4	16.6 ±41.0	16.2 ±39.8
Antigout Preparations (ATC code:M04)	8.0 ±45.4	7.6 ±44.1	7.7 ±44.2	7.1 ±42.2*
Opioids (ATC code:N02A)	3.4 ±21.0	3.7 ±20.7*	3.6 ±20.7	2.8 ±17.2*
Other Analgesics and Antipyretics (ATC code:N02B)	33.5 ±56.1	33.8 ±56.3	33.3 ±55.4	33.8 ±55.6
Antimigraine Preparations (ATC code:N02C)	3.0 ±24.3	3.2 ±25.3	3.2 ±24.6	3.6 ±27.0*
Antiepileptics (ATC code:N03A)	7.3 ±33.2	8.4 ±34.9*	7.9 ±33.4*	6.9 ±31.5
Anticholinergic Agents (ATC code:N04A)	0.9 ±14.3	1.0 ±15.9	1.1 ±16.2	0.7 ±12.8
Dopaminergic Agents (ATC code:N04B)	8.2 ±53.5	7.4 ±51.0*	7.9 ±52.7	7.6 ±51.7
Antipsychotics (First-Generation Antipsychotics Only) (ATC code:N05A)	1.1 ±12.8	1.4 ±14.7*	1.3 ±14.2	1.2 ±13.6

Anxiolytics (ATC code:N05B)	38.5 ±62.3	37.5 ±61.5*	37.4 ±61.1	34.8 ±59.0*
Hypnotics and Sedatives (ATC code:N05C)	19.6 ±42.9	21.4 ±45.3*	20.4 ±43.9*	17.9 ±40.9*
Psychostimulants, Agents Used for ADHD and Nootropics (ATC code:N06B)	5.1 ±37.6	4.6 ±35.5*	4.8 ±36.2	4.7 ±35.3
Parasympathomimetics (ATC code:N07A)	2.3 ±26.3	2.6 ±28.4	2.5 ±27.6	2.5 ±27.3
Antivertigo Preparations (ATC code:N07C)	13.3 ±42.2	13.1 ±41.6	13.3 ±41.4	13.2 ±41.5
Nasal Preparations (ATC code:R01)	16.5 ±45.4	16.3 ±45.2	16.0 ±43.8	16.4 ±45.1
Drugs for Obstructive Airway Diseases (ATC code:R03)	9.7 ±33.2	9.2 ±32.1*	9.1 ±31.6*	9.5 ±33.4
Cough and Cold Preparations (ATC code:R05)	27.0 ±50.6	26.2 ±49.8*	26.1 ±48.6	26.4 ±50.0
Antihistamines for Systemic Use (ATC code:R06)	25.7 ±48.5	25.8 ±49.0	25.9 ±48.7	25.8 ±48.2
Ophthalmologicals (ATC code:S01)	16.8 ±44.8	17.1 ±45.6	17.0 ±45.4	16.1 ±43.2

* p-value <0.05 between treatment and control groups; pair-wise comparison using chi-square test for categorical variables and t-test for continuous variables

Supplementary Table S6. The baseline characteristics of these four groups for the next-step treatment

	Model recommend (n=18,434)	As usual (n=148,556)	Randomly selected by prescription proportion (n=17,940)	Randomly selected by recommended proportion (n=14,030)
Age group				
18-29	3,653 (19.8)	32,860 (22.1)*	4,048 (22.6)*	3,231 (23.0)*
30-39	3,754 (20.4)	32,956 (22.2)*	3,967 (22.1)*	3,022 (21.5)*
40-49	4,491 (24.4)	33,947 (22.9)*	4,066 (22.7)*	3,100 (22.1)*
50-59	3,859 (20.9)	27,436 (18.5)*	3,300 (18.4)*	2,596 (18.5)*
≥60	2,677 (14.5)	21,357 (14.4)*	2,559 (14.3)*	2,081 (14.8)*
Sex, female	12,790 (69.4)	101,640 (68.4)*	12,362 (68.9)*	9,721 (69.3)*
**Type of depression**				
Major depressive disorder	7,159 (38.8)	62,562 (42.1)*	7,867 (43.9)*	5,942 (42.4)*
Dysthymic disorder	8,658 (47.0)	65,722 (44.2)*	7,662 (42.7)*	6,119 (43.6)*
Depressive disorder, NOS	2,617 (14.2)	20,272 (13.6)*	2,411 (13.4)*	1,969 (14.0)*
**Duration of illness**	2.6 ±2.4	2.5 ±2.3*	2.5 ±2.3*	2.6 ±2.4
**Comorbidity**				
Diabetes mellitus (ICD-9-CM: 250)	1,901 (10.3)	13,904 (9.4)*	1,635 (9.1)*	1,332 (9.5)*
Dyslipidemia (ICD-9-CM: 272)	3,073 (16.7)	21,980 (14.8)*	2,652 (14.8)*	2,103 (15.0)*
Asthma (ICD-9-CM: 493)	1,071 (5.8)	8,119 (5.5)	981 (5.5)	737 (5.3)*
Cancer (ICD-9-CM: 140-208, 273.0, 273.3, and V10)	758 (4.1)	5,630 (3.8)*	658 (3.7)*	516 (3.7)*
Headache (ICD-9-CM: 307.81, 346, 784.0)	7,043 (38.2)	56,267 (37.9)	6,825 (38.0)	5,400 (38.5)
Hemiplegia (ICD-9-CM: 342, 344)	161 (0.9)	1,266 (0.9)	168 (0.9)	116 (0.8)
Peripheral neurological disorder (ICD-9-CM: 351-357)	1,517 (8.2)	11,860 (8.0)	1,461 (8.1)	1,095 (7.8)
Cerebrovascular disease (ICD-9-CM: 362.34, 430-438, 781.4, 784.3, 997.0)	893 (4.8)	7,062 (4.8)	917 (5.1)	695 (5.0)
Hypertension (ICD-9-CM: 401-405)	4,124 (22.4)	30,943 (20.8)*	3,793 (21.1)*	2,972 (21.2)*
Congestive heart failure (ICD-9-CM: 402.01, 402.11, 302.91, 425, 428, 429.3)	367 (2.0)	3,024 (2.0)	382 (2.1)	264 (1.9)
Chronic kidney disease (ICD-9-CM: 403.x1, 404.x2, 585, 586, V42.0, V45.1, V56.0, V56.8)	272 (1.5)	1,909 (1.3)*	239 (1.3)	193 (1.4)
Coronary heart disease (ICD-9-CM: 410-414)	1,549 (8.4)	12,529 (8.4)	1,444 (8.0)	1,163 (8.3)
Peripheral vascular disease (ICD-9-CM: 440, 441.2, 441.4, 441.7, 441.9, 443.1- 443.9, 447.1, 557, 785.4)	351 (1.9)	2,334 (1.6)*	294 (1.6)	218 (1.6)*
Chronic pulmonary disease (ICD-9-CM: 491, 492, 494, 495, 496)	975 (5.3)	7,239 (4.9)*	873 (4.9)	651 (4.6)*
Peptic ulcer disease (ICD-9-CM: 531 – 534)	3,890 (21.1)	31,276 (21.1)	3,840 (21.4)	2,867 (20.4)
Functional gastrointestinal disorders (ICD-9-CM: 536, 564, 306.4)	7,916 (42.9)	63,609 (42.8)	7,718 (43.0)	5,933 (42.3)

Chronic liver disease (ICD-9-CM: 571.2-571.9; 456.0-456.2)	549 (3.0)	4,361 (2.9)	534 (3.0)	386 (2.8)
Rheumatological disease (ICD-9-CM: 710.0, 710.1, 710.4, 714.0, 714.1, 714.2, 714.81, 725)	322 (1.7)	2,393 (1.6)	311 (1.7)	245 (1.7)
Fibromyalgia and osteoarthritis (ICD-9-CM: 715, 729.1)	6,546 (35.5)	52,444 (35.3)	6,187 (34.5)*	4,872 (34.7)
Back pain (ICD-9-CM: 721, 722, 723, 724, 739.3,739.4, 846, 847.2)	6,643 (36.0)	52,363 (35.2)*	6,336 (35.3)	4,884 (34.8)*
Alcohol use disorder (ICD-9-CM: 291, 303.9, 305.0, 357.5, 425.5, 535.3, 571.0-571.3, V11.3)	709 (3.8)	6,639 (4.5)*	830 (4.6)*	589 (4.2)
Substance use disorder (ICD-9-CM: 292, 304, 305.2-305.9)	392 (2.1)	4,538 (3.1)*	608 (3.4)*	402 (2.9)*
Anxiety state, unspecified (ICD-9-CM: 300 without 4th or 5th digit)	147 (0.8)	1,571 (1.1)*	165 (0.9)	131 (0.9)
Panic disorder (ICD-9-CM: 300.01)	1,915 (10.4)	14,275 (9.6)*	1,692 (9.4)*	1,330 (9.5)*
Generalized anxiety disorder (ICD-9-CM: 300.02)	2,489 (13.5)	19,538 (13.2)	2,323 (12.9)	1,840 (13.1)
Phobic disorders (ICD-9-CM: 300.2)	296 (1.6)	2,343 (1.6)	279 (1.6)	224 (1.6)
Neurasthenia (ICD-9-CM: 300.5)	253 (1.4)	2,125 (1.4)	248 (1.4)	182 (1.3)
Personality disorders (ICD-9-CM: 301)	362 (2.0)	3,950 (2.7)*	531 (3.0)*	429 (3.1)*
Adjustment disorders (ICD-9-CM: 309)	1,843 (10.0)	16,599 (11.2)*	2,014 (11.2)*	1,578 (11.2)*
Obsessive-compulsive disorders (ICD-9-CM:300.3)	496 (2.7)	4,100 (2.8)	497 (2.8)	413 (2.9)
Somatoform disorders (ICD-9-CM:300.8)	419 (2.3)	3,419 (2.3)	428 (2.4)	298 (2.1)
Other anxiety disorders (ICD-9-CM:300.1, 300.6, 300.7, 300.9)	1,825 (9.9)	15,579 (10.5)*	1,850 (10.3)	1,422 (10.1)
Physiological malfunction arising from mental factors (ICD-9-CM:306)	1,108 (6.0)	8,951 (6.0)	1,087 (6.1)	783 (5.6)
Sleep disorders (ICD-9-CM:307.4, 780.5)	11,438 (62.0)	92,674 (62.4)	11,221 (62.5)	8,660 (61.7)
Eating disorders (ICD-9-CM: 307.1, 307.5)	254 (1.4)	2,200 (1.5)	270 (1.5)	224 (1.6)
**Number of outpatient visits**				
Psychiatry	5.2 ±3.9	5.2 ±4.0	5.3 ±4.0	5.1 ±4.0*
Family medicine	3.6 ±3.8	3.6 ±3.8	3.6 ±3.8	3.5 ±3.8
Internal medicine	3.5 ±3.8	3.4 ±3.8	3.5 ±3.8	3.4 ±3.7*
Dermatology	0.9 ±1.9	0.9 ±2.0	0.9 ±1.9	0.9 ±2.0
Neurology	0.8 ±2.1	0.8 ±2.1	0.8 ±2.1	0.8 ±2.0
Obstetrics and gynecology	1.6 ±2.7	1.6 ±2.7	1.6 ±2.8	1.6 ±2.7
Ophthalmology	1.1 ±2.1	1.0 ±2.0*	1.0 ±2.1*	1.0 ±2.1
Orthopedics	0.9 ±2.1	0.9 ±2.1	0.9 ±2.0	0.9 ±2.0
Otolaryngology	1.9 ±2.9	1.8 ±2.8*	1.8 ±2.8*	1.8 ±2.8*
Rehabilitation	0.5 ±1.7	0.5 ±1.6	0.5 ±1.6	0.4 ±1.6*
Surgery	0.7 ±1.8	0.7 ±1.8	0.7 ±1.8	0.7 ±1.8
Other specialties	2.0 ±3.0	1.9 ±2.9*	1.9 ±2.9*	1.9 ±2.9*
Transitional Chinese medicine	2.4 ±3.4	2.3 ±3.3*	2.3 ±3.3*	2.4 ±3.4

Dentistry	1.7 ±2.4	1.6 ±2.4*	1.6 ±2.4*	1.6 ±2.4
**Number of emergency medicine**	0.6 ±1.3	0.7 ±1.5*	0.7 ±1.5*	0.7 ±1.5*
**Inpatient treatment**				
Psychiatry	548 (3.0)	6,164 (4.1)*	796 (4.4)*	563 (4.0)*
Neurology	251 (1.4)	2,021 (1.4)	260 (1.4)	189 (1.3)
Internal medicine	1,224 (6.6)	10,414 (7.0)	1,279 (7.1)	912 (6.5)
Surgery	763 (4.1)	6,607 (4.4)	830 (4.6)*	639 (4.6)
Obstetrics and gynaecology	355 (1.9)	3,050 (2.1)	392 (2.2)	296 (2.1)
Orthopedics	383 (2.1)	3,447 (2.3)*	421 (2.3)	327 (2.3)
Urologist	144 (0.8)	1,155 (0.8)	163 (0.9)	114 (0.8)
Other specialties	520 (2.8)	3,825 (2.6)*	486 (2.7)	360 (2.6)
**Medication use, days**				
Stomatological Preparations (ATC code:A01)	4.5 ±27.4	4.5 ±27.1	4.5 ±27.6	4.6 ±27.3
Antacids (ATC code:A02A)	36.4 ±61.5	37.5 ±61.8*	37.9 ±62.0*	36.5 ±60.9
Drugs for Peptic Ulcer and Gastro-esophageal Reflux Disease (ATC code:A02B)	23.2 ±56.7	23.4 ±56.6	23.8 ±56.8	23.4 ±56.3
Drugs for Functional Gastrointestinal Disorders (ATC code:A03A)	31.9 ±61.7	33.3 ±63.1*	33.5 ±62.7*	32.8 ±63.2
Belladonna and Derivatives, Plain (ATC code:A03B)	11.6 ±39.9	11.5 ±38.3	11.4 ±37.4	11.1 ±37.3
Propulsives (ATC code:A03F)	29.6 ±63.3	30.8 ±65.2*	30.9 ±65.1	30.1 ±64.0
Antiemetics and Antinauseants (ATC code:A04)	7.5 ±34.2	8.2 ±35.4*	8.1 ±34.7	8.1 ±35.6
Bile and Liver Therapy (ATC code:A05)	7.0 ±35.7	6.6 ±34.5	6.3 ±33.7	6.5 ±34.6
Drugs for Constipation (ATC code:A06)	16.9 ±48.4	17.8 ±50.4*	18.4 ±51.2*	17.2 ±48.2
Antidiarrheals, Intestinal Antiinflammatory/Antiinfective Agents (ATC code:A07)	15.6 ±50.6	16.0 ±50.6	16.2 ±51.2	15.9 ±49.4
Digestives, Incl. Enzymes (ATC code:A09)	9.1 ±40.8	9.2 ±40.3	9.6 ±41.6	8.3 ±37.0
Drugs Used In Diabetes (ATC code:A10)	16.8 ±66.6	14.8 ±62.5*	14.4 ±61.6*	15.4 ±63.4
Vitamins (ATC code:A11)	11.2 ±39.1	10.6 ±38.0	10.5 ±37.4	10.4 ±37.6
Mineral Supplements (ATC code:A12)	8.7 ±47.2	8.3 ±45.4	8.3 ±45.2	8.6 ±46.6
Antithrombotic Agents (ATC code:B01)	14.5 ±52.6	13.7 ±51.1*	13.8 ±51.3	13.4 ±50.3
Antihemorrhagics (ATC code:B02)	13.7 ±49.0	12.9 ±46.2*	13.2 ±46.5	12.8 ±45.4
Antianemic Preparations (ATC code:B03)	10.4 ±47.7	9.8 ±45.0	10.7 ±48.1	10.0 ±45.6
Blood Substitutes and Perfusion Solutions (ATC code:B05)	13.1 ±39.6	13.2 ±37.9	13.3 ±37.1	13.0 ±37.8
Other Hematological Agents (ATC code:B06)	17.7 ±52.1	18.2 ±52.9	18.1 ±52.5	17.5 ±51.6
Cardiac Therapy (ATC code:C01)	8.3 ±40.5	8.1 ±40.1	8.0 ±39.6	7.7 ±39.0
Antihypertensives (ATC code:C02)	6.8 ±46.5	7.7 ±49.2*	7.7 ±49.2	7.7 ±49.4
Diuretics (ATC code:C03)	9.8 ±44.0	8.7 ±40.4*	8.5 ±39.3*	9.0 ±40.7

Peripheral Vasodilators (ATC code:C04)	11.9 ±43.8	11.9 ±44.4	12.9 ±47.1*	11.5 ±43.7
Vasoprotectives (ATC code:C05)	8.1 ±41.0	8.1 ±40.4	8.3 ±41.3	8.3 ±40.4
Beta Blocking Agents (ATC code:C07)	33.5 ±60.8	35.9 ±64.5*	35.7 ±63.2*	35.6 ±63.7*
Calcium Channel Blockers (ATC code:C08)	19.7 ±58.3	19.3 ±58.1	19.7 ±58.8	19.9 ±58.3
Agents Acting On The Renin-Angiotensin System (ATC code:C09)	20.4 ±66.4	19.2 ±65.0*	19.3 ±64.6	19.7 ±65.3
Lipid Modifying Agents (ATC code:C10)	18.8 ±62.5	16.1 ±57.9*	16.5 ±58.5*	15.8 ±57.2*
Antifungals for Dermatological Use (ATC code:D01)	9.8 ±37.4	10.0 ±37.6	10.0 ±37.5	9.7 ±37.0
Emollients and Protectives (ATC code:D02)	3.6 ±26.4	3.3 ±24.3	3.5 ±26.1	3.7 ±26.5
Antipsoriatics (ATC code:D05)	3.1 ±29.6	3.4 ±31.6	3.3 ±30.7	3.2 ±30.5
Antibiotics and Chemotherapeutics for Dermatological Use (ATC code:D06)	7.5 ±31.1	7.2 ±29.3	7.4 ±28.8	7.6 ±30.2
Corticosteroids, Dermatological Preparations (ATC code:D07)	22.3 ±55.1	22.4 ±55.2	22.6 ±56.1	22.4 ±54.8
Anti-Acne Preparations (ATC code:D10)	9.7 ±42.3	10.0 ±42.0	10.6 ±43.0	10.3 ±42.3
Gynecological Antiinfectives and Antiseptics (ATC code:G01)	13.4 ±46.8	13.7 ±46.1	13.9 ±46.7	13.4 ±45.2
Sex Hormones and Modulators Of The Genital System (ATC code:G03)	20.0 ±61.7	19.7 ±61.5	20.2 ±61.9	20.1 ±61.7
Urologicals (ATC code:G04)	9.7 ±40.7	10.1 ±40.7	10.3 ±41.3	10.0 ±40.4
Corticosteroids for Systemic Use (ATC code:H02)	19.7 ±57.4	18.8 ±55.1*	19.6 ±56.8	19.0 ±55.4
Thyroid Therapy (ATC code:H03)	4.3 ±32.4	4.6 ±33.7	4.8 ±34.9	4.6 ±33.6
Antibacterials for Systemic Use (ATC code:J01)	27.6 ±50.5	27.8 ±49.1	28.5 ±49.8	27.5 ±49.1
Antimycotics for Systemic Use (ATC code:J02)	2.2 ±19.5	2.4 ±20.2	2.4 ±20.0	2.5 ±20.6
Antivirals for Systemic Use (ATC code:J05)	20.4 ±57.4	19.0 ±54.8*	19.5 ±55.3	19.5 ±56.2
Vaccines (ATC code:J07)	7.7 ±32.8	8.4 ±33.6*	8.9 ±34.6*	8.8 ±35.5*
Immunostimulants (ATC code:L03)	2.4 ±26.3	2.3 ±25.6	2.1 ±24.8	2.5 ±27.3
Antiinflammatory and Antirheumatic Products (ATC code:M01)	43.7 ±67.8	44.4 ±68.7	45.1 ±69.0*	43.8 ±67.8
Topical Products for Joint and Muscular Pain (ATC code:M02)	16.8 ±56.8	17.1 ±56.6	16.9 ±56.5	16.9 ±56.3
Muscle Relaxants (ATC code:M03)	29.0 ±62.3	28.7 ±61.4	29.6 ±62.8	28.3 ±59.8
Antigout Preparations (ATC code:M04)	7.7 ±44.9	7.0 ±42.3*	7.5 ±44.2	6.7 ±41.0*
Opioids (ATC code:N02A)	12.0 ±50.7	11.3 ±48.1	12.0 ±49.7	11.6 ±48.9
Other Analgesics and Antipyretics (ATC code:N02B)	35.3 ±56.7	36.2 ±58.6	36.6 ±58.2*	36.5 ±57.8
Antimigraine Preparations (ATC code:N02C)	8.2 ±46.5	8.9 ±48.7	9.0 ±49.2	9.0 ±48.9

Antiepileptics (ATC code:N03A)	35.0 ±66.3	36.1 ±67.8*	38.3 ±69.6*	35.8 ±66.7
Anticholinergic Agents (ATC code:N04A)	2.9 ±24.5	5.0 ±33.1*	5.1 ±33.6*	4.8 ±33.1*
Dopaminergic Agents (ATC code:N04B)	3.5 ±30.9	3.8 ±32.5	3.9 ±33.2	3.9 ±32.6
Antipsychotics (First-Generation Antipsychotics Only) (ATC code:N05A)	5.2 ±29.7	7.3 ±35.9*	7.8 ±36.7*	6.5 ±33.3*
Anxiolytics (ATC code:N05B)	73.2 ±73.5	76.6 ±81.0*	77.6 ±81.2*	74.7 ±79.4
Hypnotics and Sedatives (ATC code:N05C)	64.4 ±68.1	67.8 ±80.0*	69.2 ±79.8*	63.9 ±76.8
Psychostimulants, Agents Used for ADHD and Nootropics (ATC code:N06B)	6.3 ±39.6	6.3 ±39.4	6.5 ±40.0	6.9 ±40.9
Parasympathomimetics (ATC code:N07A)	2.5 ±25.5	2.6 ±26.0	2.7 ±26.7	3.2 ±29.3*
Antivertigo Preparations (ATC code:N07C)	16.2 ±45.4	16.2 ±45.0	16.3 ±44.7	15.1 ±42.7*
Nasal Preparations (ATC code:R01)	16.3 ±45.1	16.2 ±44.4	16.3 ±45.1	16.6 ±45.7
Drugs for Obstructive Airway Diseases (ATC code:R03)	26.8 ±68.3	25.5 ±65.8*	26.0 ±67.1	25.2 ±64.8*
Cough and Cold Preparations (ATC code:R05)	35.3 ±62.1	34.4 ±61.2*	34.4 ±61.2	33.1 ±58.6*
Antihistamines for Systemic Use (ATC code:R06)	28.7 ±52.3	27.8 ±50.9*	28.4 ±51.3	27.0 ±49.4*
Ophthalmologicals (ATC code:S01)	21.0 ±54.2	20.1 ±52.3*	20.4 ±52.7	20.3 ±52.3
**History of previous antidepressant regimen in the past year**				
**Psychotropic agent use in the past year (days)**				
Amitriptyline	4.6 ±29.6	5.7 ±33.9*	5.1 ±31.1	5.2 ±31.4
Bupropion	12.2 ±51.0	13.9 ±54.1*	13.0 ±51.9	13.5 ±54.1*
Citalopram	17.7 ±54.5	16.2 ±53.1*	16.3 ±52.5*	15.6 ±51.7*
Doxepin	3.2 ±24.3	3.6 ±26.4*	3.0 ±24.3	3.2 ±24.3
Duloxetine	9.7 ±44.6	10.3 ±46.7	10.0 ±46.0	10.5 ±46.9
Escitalopram	23.3 ±64.3	20.4 ±61.1*	21.2 ±62.7*	26.3 ±69.3*
Fluoxetine	29.5 ±68.5	28.8 ±67.2	30.5 ±69.1	28.0 ±65.9*
Fluvoxamine	7.8 ±42.5	9.8 ±47.2*	8.9 ±44.5*	7.4 ±40.4
Imipramine	8.4 ±37.7	9.2 ±39.9*	8.7 ±38.2	8.4 ±38.1
Milnacipran	4.3 ±36.5	5.0 ±39.5*	4.4 ±36.7	4.4 ±36.7
Mirtazapine	18.1 ±56.8	21.9 ±62.1*	23.8 ±64.7*	20.4 ±60.3*
Moclobemide	5.5 ±34.6	5.1 ±32.5	4.7 ±31.6*	5.1 ±32.7
Paroxetine	20.7 ±56.5	21.3 ±57.5	22.9 ±60.1*	19.7 ±54.8
Sertraline	30.1 ±70.8	29.7 ±71.6	31.3 ±73.5	30.1 ±72.2
Trazodone	32.6 ±67.1	35.6 ±72.0*	36.0 ±71.8*	34.2 ±70.4*
Venlafaxine	23.8 ±64.7	21.7 ±61.4*	22.1 ±62.0*	19.9 ±58.4*
Lamotrigine	1.6 ±20.7	1.9 ±22.8	1.9 ±22.9	2.2 ±24.6*
Lithium	1.2 ±16.2	1.5 ±18.6*	1.9 ±20.7*	1.4 ±17.1
Valproic Acid	4.6 ±30.3	5.2 ±32.0*	6.0 ±34.4*	5.3 ±32.5*
Amisulpride	1.8 ±24.1	2.0 ±25.3	2.0 ±25.3	2.1 ±25.6

Aripiprazole	3.8 ±35.4	3.5 ±33.6	4.0 ±36.4	4.4 ±38.2
Olanzapine	3.0 ±31.4	3.6 ±33.7	3.9 ±35.5*	3.1 ±31.3
Quetiapine	6.4 ±30.6	8.4 ±37.3*	9.3 ±38.6*	8.9 ±38.4*
Risperidone	4.7 ±40.9	6.0 ±46.1*	6.2 ±46.8*	6.3 ±47.4*
Zotepine	1.9 ±23.9	2.1 ±24.8	2.1 ±24.2	2.1 ±24.9
**Characteristics of the latest antidepressant regimen**				
**Number of failed treatment episodes**	98.3 ±143.1	99.6 ±146.1	92.4 ±141.6*	98.4 ±145.1
1	6,983 (37.9)	55,178 (37.1)*	6,594 (36.8)*	5,267 (37.5)*
2	4,103 (22.3)	29,087 (19.6)*	3,572 (19.9)*	2,776 (19.8)*
3-4	3,719 (20.2)	28,290 (19.0)*	3,455 (19.3)*	2,644 (18.8)*
≥ 5	3,629 (19.7)	36,001 (24.2)*	4,319 (24.1)*	3,343 (23.8)*

* p-value <0.05 between treatment and control groups; pair-wise comparison using chi-square test for categorical variables and t-test for continuous variables

1. WHO. ATC/DDD Index 20192019.
2. Giakoumatos CI, Osser D. The psychopharmacology algorithm project at the Harvard South Shore Program: an update on unipolar nonpsychotic depression. *Harv. Rev. Psychiatry.* 2019;27:33-52.
3. MacQueen G, Santaguida P, Keshavarz H, et al. Systematic review of clinical practice guidelines for failed antidepressant treatment response in major depressive disorder, dysthymia, and subthreshold depression in adults. *Can. J. Psychiatry.* 2017;62:11-23.
4. Imai K, Ratkovic M. Covariate balancing propensity score. *J R Stat Soc Series B Stat Methodol.* 2014;76:243-63.
5. Cole SR, Hernán MA. Constructing inverse probability weights for marginal structural models. *Am. J. Epidemiol.* 2008;168:656-64.
