# Supplementary figures and images for: Validation of Machine Learning-Based Individualized Treatment for Depressive Disorder Using Target Trial Emulation
Source: J Pers Med. 2021 Dec 7;11(12):1316. doi: 10.3390/jpm11121316 (PMC8706481; doi:10.3390/jpm11121316)

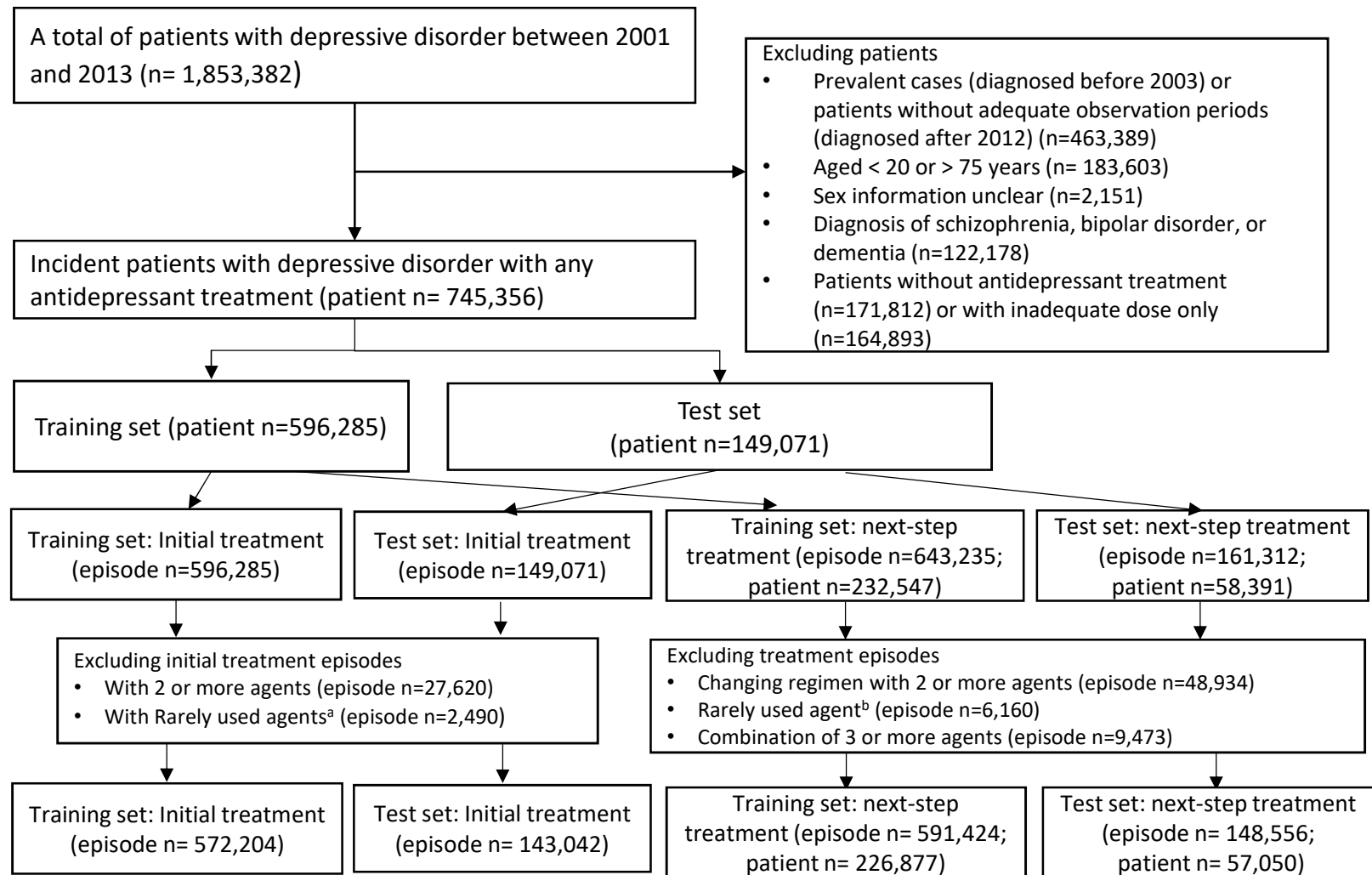

Supplement: Supplementary file 1 [file jpm-11-01316-s001.zip › Supplementary Fig S1.pdf]
